# Molecular Insight into the Role of Vitamin D in Immune-Mediated Inflammatory Diseases

**DOI:** 10.3390/ijms26104798

**Published:** 2025-05-16

**Authors:** Christiano Argano, Alessandra Torres, Valentina Orlando, Virginia Cangialosi, Dalila Maggio, Chiara Pollicino, Salvatore Corrao

**Affiliations:** 1 Department of Internal Medicine, National Relevance and High Specialization Hospital Trust ARNAS Civico, Di Cristina, Benfratelli, 90127 Palermo, Italy; salvatore.corrao@unipa.it; 2 Department of Health Promotion Sciences, Maternal and Infant Care, Internal Medicine and Medical Specialties (PROMISE), University of Palermo, 90133 Palermo, Italy; alestor@live.it (A.T.); valeorlando96@gmail.com (V.O.); virginiacangialosi@gmail.com (V.C.); maggio.dalila@gmail.com (D.M.); chiarapollicino94@gmail.com (C.P.)

**Keywords:** innate immunity, adaptive immunity, immune-mediated inflammatory disorders, asthma, atopic dermatitis, rheumatoid arthritis, psoriasis, thyroid diseases, infectious diseases, systemic lupus erythematosus

## Abstract

In the last decades, it has become increasingly evident that the role of vitamin D extends beyond the regulation of calcium homeostasis and the maintenance of bone health. A significant extraskeletal function of vitamin D is its role in modulating the immune system, particularly highlighted in the context of immune-mediated inflammatory diseases, where correlations between vitamin D status and genetic variations in the vitamin D receptor have been observed about the incidence and severity of these conditions. Additionally, different studies have reported the existence of immunomodulatory effects of vitamin D, particularly the effects of vitamin D on dendritic cell function, maturation, cytokine production, and antigen presentation, and that its deficiency may be associated with a sub-inflammatory state. In this sense, different clinical trials have been conducted to assess the therapeutic efficacy of vitamin D in different immune-mediated inflammatory disorders, including asthma, atopic dermatitis (AD), rheumatoid arthritis (RA), psoriasis, thyroid diseases, infectious diseases, and systemic lupus erythematosus (SLE). This review will provide a comprehensive overview of the current understanding of the molecular mechanisms underlying vitamin D’s immunomodulatory properties, its role, and innovative therapeutic applications in patients with immune-mediated inflammatory diseases.

## 1. Introduction

In recent years, attention has been paid to the role of vitamin D in different fields. vitamin D is a fat-soluble prohormone critical in various physiological processes, particularly in bone metabolism. In addition to its well-established role in promoting calcium absorption and bone health, vitamin D is implicated in many extraskeletal functions. Its functions encompass endocrine, autocrine, and paracrine mechanisms essential for overall health [1]. Different studies have increasingly highlighted the correlation between vitamin D deficiency and several pathological conditions. Some evidence shows its systemic effects, such as regulation of cell differentiation, proliferation, activation and death, xenobiotic detoxification, antioxidant and neuroprotective actions, antimicrobial defense, immunoregulation of both innate and adaptive immunity, and anti-inflammatory and anti-neoplastic effects in many cells and tissues [2,3,4,5,6]. In this sense, serum vitamin D levels are related to the incidence of chronic inflammatory diseases such as cardiovascular diseases, diabetes, insulin resistance, metabolic syndrome, autoimmune diseases, allergic disorders, infectious diseases, and tumors. Particularly, vitamin D may serve as a significant predisposing factor in diabetes pathogenesis, wherein dysregulation of its signaling pathways elevates susceptibility to autoimmune dysregulation and macrovascular and microvascular complications [7,8,9,10,11,12,13,14,15,16,17].

**Figure 1:**
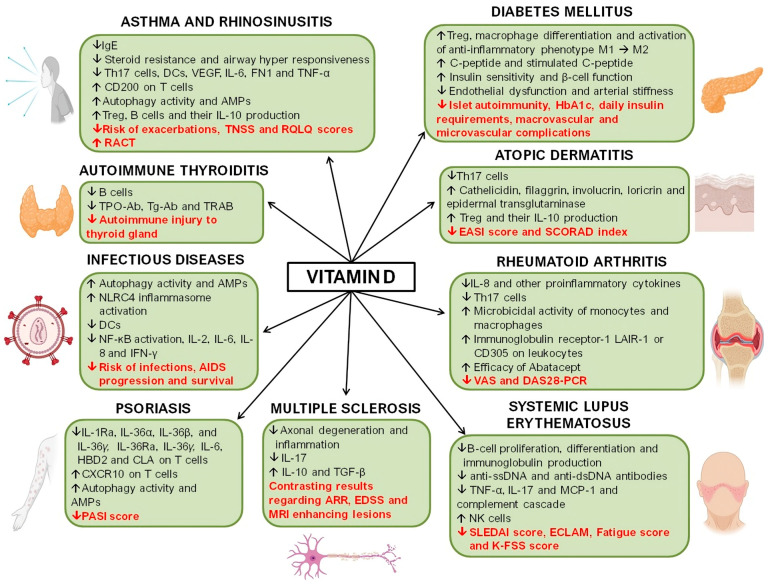
The immune-mediated and anti-inflammatory actions of vitamin D from bench to bedside.

The recent COVID-19 pandemic has further emphasized the potential therapeutic role of vitamin D in managing infectious diseases. Emerging studies suggest that adequate levels of vitamin D favorably impact immune responses, influencing the severity of COVID-19 infections. Insufficient vitamin D levels have been linked to worse health outcomes in patients suffering from COVID-19, raising concerns about the implications of vitamin D deficiency during the pandemic [18,19,20]. This growing body of evidence underscores the importance of monitoring and potentially supplementing vitamin D to enhance health and mitigate the risk of various diseases. Some studies have reported the immunomodulatory effects of vitamin D, particularly the effects of vitamin D on dendritic cell (DC) function, maturation, cytokine production, and antigen presentation, and that its deficiency may be associated with a sub-inflammatory state [3,21,22]. Moreover, immune cells express the vitamin D receptor (VDR) and a vitamin D activating enzyme, indicating that these cells can produce and respond to activated vitamin D, suggesting that its deficiency may significantly impact inflammatory disorders [23,24,25,26] (Figure 1).

Given this background, an extensive search of SCOPUS, PubMed, and CENTRAL was performed using the following string: “((vitamin D) or (calciferol) or (cholecalciferol) or (calcifediol) or (calcitriol) or (ergocholecalciferol)) AND ((autoimmune diseases) OR (autoinflammatory disorders) OR (immune system disorders) OR (immunological disorders) OR (chronic inflammatory diseases)) AND (systematic review [pt] or meta-analysis [pt]) and 2015:2025 [dp])”. The search string retrieved 301 manuscripts. The hand-searching of principal generalist, human nutrition, and basic research journals was also carried out [27,28,29]. Two authors (V.O. and A.T.) independently reviewed the retrieved articles’ titles, abstracts, and full texts to determine their potential inclusion. Any disagreements were resolved via discussion with the third author (S.C.). Manuscripts regarding the role of vitamin D in immune-mediated inflammatory diseases were extracted for this review. This review explores the molecular basis, the role, and the innovative therapeutic applications of vitamin D in immune-mediated inflammatory diseases.

## 2. Vitamin D Metabolism

**Figure 2:**
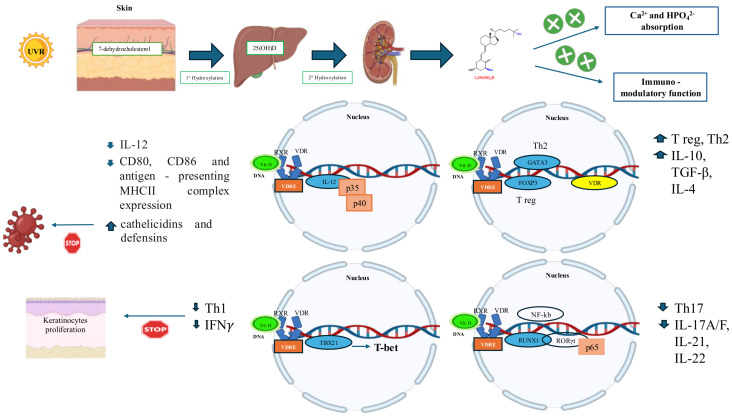
Synthesis and mechanism of action of vitamin D on bone metabolism and, in particular, on innate and adaptive immunity cells. Transcription factors FOXP3 (Forkhead box P3), GATA3, TBX21 (T-bet), RUNX1, RORγt, and NF-kb (protein complex functioning as a transcription factor) with its isoform p65. P35 and P40 are subunits of IL-12. VDRE is a type of DNA sequence found in the promoter region of vitamin D-regulated genes.

Originally classified as a vitamin, vitamin D is now recognized as a prohormone essential for multiple physiological functions. It is a liposoluble compound found in two inactive forms: cholecalciferol (vitamin D3), synthesized in the skin upon UVB exposure and found in animal-based foods (particularly fatty fish such as herring, mackerel, sardines, and salmon), and ergocalciferol (vitamin D2), derived from UV-exposed fungi and yeasts that, when exposed to UV light, produce vitamin D2 from a precursor called ergosterol, which is present in their cell membranes, and primarily ingested through fortified foods [3,14,30]. Despite structural differences in their side chains, D2 and D3 share the same metabolic pathway. Upon absorption in the small intestine, they are incorporated into chylomicrons and transported to the liver, where they bind to vitamin D Binding Protein (DBP) and are taken up by hepatocytes. Hepatic 25-hydroxylase (CYP2R1) converts them into 25-hydroxyvitamin D (25(OH)D or calcifediol), the main circulating form [4]. In the mitochondria of the proximal convoluted tubule cells in the kidneys, 25(OH)D undergoes further hydroxylation by mitochondrial 1α-hydroxylase (CYP27B1) to become the biologically active 1α,25-dihydroxyvitamin D (1α,25(OH)_2_D or calcitriol), or by 24-hydroxylase (CYP24A1) to form 24,25(OH)_2_D, a metabolite implicated in bone repair and autoregulation of the vitamin D pathway [4,14]. The expression of CYP24A1 is upregulated by high calcitriol levels and downregulated by low calcium/phosphate or elevated PTH, reflecting tight feedback control. A secondary metabolite, 24,25(OH)2D, is mainly produced when calcitriol levels are high, with a key role in regulating bone metabolism and vitamin D. High levels of calcitriol activate the expression of CYP24A1, which increases the synthesis of 24,25(OH)2D and promotes the degradation of calcitriol. Low levels of calcium and phosphate inhibit CYP24A1 and promote the production of calcitriol instead of 24,25(OH)2D. Parathyroid Hormone (PTH) inhibits CYP24A1 and stimulates the production of calcitriol. 24R,25(OH)2D plays a key role in modulating bone metabolism and promoting bone repair, particularly in fractures. It can degrade calcitriol, reducing levels of active vitamin D and protecting against hypercalcemia. It has also recently been suggested that it may play a role in regulating the immune response [26]. Calcitriol exerts its effects via the vitamin D receptor (VDR), a nuclear receptor that forms heterodimers with Retinoid X Receptor (RXR) to regulate gene expression through vitamin D Response Elements (VDREs). The VDR is widely expressed in various body tissues, including the intestine, pancreas, kidney, bone, bronchi, skin, osteoblasts, chondrocytes, endocrine glands, reproductive tissues, and immune cells. As a result, the VDR-RXR complex interacts with Vitamin D Response Elements (VDREs) located in the promoter regions of specific target genes, thereby positively or negatively regulating their expression by recruiting VDR-interacting nuclear proteins (coregulators) to the transcriptional preinitiation complex [31]. After ligand binding, the repositioning of helix 12 to the COOH terminus of the VDR ligand-binding domain, known as the ligand-dependent Activation Function 2 (AF2), confers a major conformational change in the three-dimensional structure of the VDR. This activation step appears necessary for the recruitment of motor proteins by the VDR [26,32], which are responsible for the rapid translocation of the cytoplasmic VDR along microtubules to the nucleus. Point mutations in the two nuclear localizing signals result in defective cytoplasmic-nuclear translocation and the type II vitamin D rickets phenotype [33,34]. Selective association between VDR and its protein partner, RXR, involves dimerization surfaces in three domains of the VDR molecule and induces a VDR conformation that is essential for the transactivating function of VDR. An interaction between the ligand binding and heterodimerization domains has been suggested by two naturally occurring mutations (I314S and R391C) in the LBD of VDR, which confer the vitamin D resistance phenotype by significantly impairing both VDR-RXR heterodimerization and ligand retention [35]. Recently discovered VDR interactions with nuclear coregulatory molecules provide new mechanistic insights into VDR-mediated positive and negative modulation of transcription [36]. Recent studies suggest a bifunctional role for the VDR co-modulator NCoA62/Skip: it can promote transcriptional activation or repression in a cell-specific manner, depending on the expression of coregulatory molecules. The coactivator p300 and the corepressors NCoR and SMRT interact with the same NH2-terminal region of the Skip molecule. The relative expression levels of the nuclear corepressor NCoR and the coactivator CBP/p300 in CV-1 and P19 cells determine whether Skip activates or represses VDR/RXR-dependent transcription [37]. More importantly, the 1,25(OH)2D-VDR complex selectively induces the expression of genes from two important families of coregulators, TIF2 among the p160 coactivators and SMRT among the corepressors [38]. Since SRC1/TIF2 ratios have been shown to influence important cellular functions, such as energy metabolism in mouse adipose tissue [39], the apparent cellular specificity of the 1,25(OH)2D-VDR complex in inducing gene transcription, and possibly TIF2 and SMRT protein abundance, suggests that 1,25(OH)2D itself may modulate the transcriptional competence of target cells. In addition to Skip, a novel ATP-dependent chromatin remodeling complex containing the Williams syndrome transcription factor potentiates ligand-induced VDR action in transactivation and gene repression. NCoA62/Skip also mediates a link between transcriptional regulation by the VDR (and other nuclear receptors) and RNA splicing by the spliceosome. Skip physically interacts with components of the splicing machinery and with nuclear matrix-associated proteins. Indeed, the expression of a dominant-negative Skip interfered with the proper splicing of 1,25(OH)2D-VDR transactivation-derived transcripts [40]. In conclusion, complex cell-specific and promoter-specific interactions of VDR nuclear coregulators are responsible for VDR regulation of vitamin D-responsive gene expression, including VDR coactivator and corepressor molecules. This intricate mechanism underlies the pleiotropic effects of vitamin D on numerous biological processes throughout the body [5,26,41] (Figure 2).

Further clarification is needed on the three forms of vitamin D, which are similar but not identical. The presence of vitamin D epimers in clinical trials is important because some methods of measuring 25(OH)D may include its epimers, potentially overestimating the levels of active vitamin D. The best-known epimers are:

– 1α,25(OH)2D (calcitriol), which is the biologically active form of vitamin D. It is essential for calcium metabolism and the regulation of many cellular processes. It is associated with osteoporosis, rickets, hypocalcemia, immune dysfunction, and some autoimmune diseases.
– 3-epi-1α,25(OH)2D (3-epi-calcitriol), which has a different configuration at carbon 3 of ring A. It has reduced affinity for the VDR and reduced biological effects. Its role is not fully understood, but it may be less calcium-affecting than calcitriol, with therapeutic implications in conditions requiring control of calcium metabolism. Do not alter the core message.
– 24R,25(OH)D, which is a less well-understood metabolite of vitamin D involved in bone repair and regulation of vitamin D metabolism. It has been linked to a protective role in certain bone diseases and the regulation of vitamin D excess [30].

## 3. Molecular Effects of Vitamin D on Immune Cells

Recent studies have shown that immune cells express VDR and produce enzymes when stimulated by vitamin D, so vitamin D deficiency may impact inflammatory diseases [26].

Monocytes and macrophages recognize bacteria, fungi, and viruses via Toll-Like Receptors (TLRs) on their surface. This activates VDR and CYP27B1 [26]. Vitamin D’s role in host defense is via an autocrine pathway in monocytes and macrophages, stimulated by TLRs2/1 and TLR4, Interferon-γ (IFN-γ), or CD40 [31,32]. Activation of these receptors initiates a signaling cascade responsible for the microsomal-mediated upregulation of cytoplasmic levels of VDR and CYP27B1, resulting in the conversion of 25(OH)D to 1,25(OH)2D. This results in calcitriol peaks in the cytosol, which drive signaling and calcitriol/VDR complexes, leading to the expression of multiple genes that modulate monocyte/macrophage function during infection. The circulating hormone calcitriol does not enter target cells at significant levels. This is important for balanced intracellular signaling, which is critical for immune cell functions [42]. Levels of vitamin D in the blood are tightly regulated to prevent too much VDR signaling. This is achieved through a metabolic feedback loop involving conversion by the inactivating enzyme 24-hydroxylase (CYP24A1) to inactive calcitroic acid, which is excreted in the bile. Immune cells express CYP27B1, which allows them to activate functional VDR signaling pathways by converting inactive vitamin D to an active form [33].

The heterodimer VDR-RXR binds to DNA and induces the assembly of cathelicidin antimicrobial peptide (cAMP), β-defensin 2 (HBD2), and other antimicrobial peptides (AMPs) with bactericidal and viricidal activity [26,34,35]. AMPs have dual roles in innate immune defense: antimicrobial activity and chemokine, cytokine, and ROS induction. CAMP is found in keratinocytes and cells of the adaptive immune system and helps with new blood vessel formation, wound healing, chemokine expression, and the movement of various immune cells, especially in response to mycobacterial organisms such as tuberculosis and leprosy, and other types of bacteria. The vitamin D pathway is the only one capable of regulating cAMP expression in keratinocytes by two mechanisms: direct regulation of cAMP and epigenetic action (histone acetylation) on VDRE, which is located in the promoter region of the cAMP gene [36,37]. Calcitriol activates the NF-kB transcription factor, which regulates NOD2 expression and muramyl dipeptide binding. The muramyl dipeptide binding is a peptidoglycan found in Gram-negative bacteria that promotes the transcription of DEFB4, the gene for β-defensin 2, via Nuclear Factor Kappa light chain (NF-κB) [26,37]. Some authors have discovered that transforming growth factor-1 (TGF-1) interferes with the signaling pathway activated by vitamin D, by reducing VDR expression and suppressing NF-kB transcription factor activity [38]. This reduces the expression of genes involved in host defense and impairs airway epithelial cells’ ability to fight respiratory pathogens. Vitamin D induces the transcription of Atg-5 and Beclin-1, which promote autophagy via the induction of cAMP and its downstream factors (p38, ERK, and C/EBPβ) [26,37]. Autophagy is important in the antimicrobial response in several immune-mediated inflammatory diseases, such as asthma, atopic dermatitis, rheumatoid arthritis and psoriasis, and in infectious diseases. In this context, vitamin D counteracts steroid resistance by inhibiting the gene for a steroid; therefore, lower serum vitamin D levels are associated with higher corticosteroid requirements, which has important implications, particularly for asthma [3,39,40,41,43].

**Figure 3:**
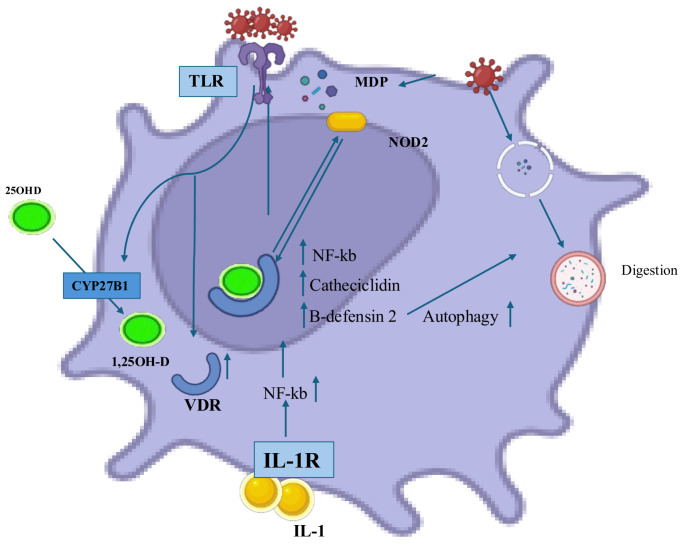
Mechanism of action of vitamin D on monocytes and macrophages. TLRs expressed on monocytes’ and macrophages’ surfaces recognize components of bacteria, fungi, and viruses, upregulating the expression of VDR and CYP27B1. CYP27B1 metabolizes 25-hydroxyvitamin D into calcitriol and binds to VDR. The activated VDR forms a heterodimer with RXR, which induces the synthesis of cathelicidin, β-defensin 2 that promotes bacterial and viral autophagy. 25(OH)D: 25-hydroxyvitamin D; 1,25(OH)2D: 1-25-Dihydroxyvitamin D, NF-kb: Nuclear Factor Kappa light chain-enhancer of activated B cells; IL-1R: interleukin-1 receptor; NOD2: Nucleotide-binding oligomerization domain-containing protein 2; TLR: Toll-Like Receptor; VDR: vitamin D receptor.

Vitamin D, mediated by transforming growth factor-β (TGF-β), plays a role in the inflammatory response of cells of the monocyte/macrophage lineage. Macrophages contain 1α-hydroxylase and are therefore capable of autocrine or paracrine activation of vitamin D. In the context of local dermal inflammation, CD69 overexpression has been reported in systemic lupus erythematosus (SLE), Graves’ disease (GD), and Hashimoto’s thyroiditis (HT). The combination of TGF-β and 1,25(OH)2D has been shown to reinduce the differentiation antigen CD69 in monocytic cells [3] (Figure 3).

Calcitriol downregulates MHC class II and co-stimulates CD40, CD80, and CD86 in dendritic cells (DCs), activating T cells. It also prevents the maturation of DCs that express VDR, thereby inhibiting excessive inflammation in response to infectious diseases [44,45].

B and T cells express VDR and CYP27B1.VDR mRNA is expressed at low levels in B cells, but these cells may be capable of autocrine/intracrine signaling, and the receptors can be upregulated by stimulation with 1,25(OH)2D [46]. A recent study showed that calcitriol suppresses the differentiation of B cells and their maturation into memory B cells and plasma cells [25,26]. It may also inhibit B cell proliferation by upregulating mRNA at the level of p27, thereby stopping the cell cycle entry of previously cycling B cells.

It also has inhibitory effects on plasma cell differentiation and immunoglobulin (Ig) production, which has important implications for the role of vitamin D in some pathologies such as asthma, autoimmune thyroiditis, and SLE. Vitamin D suppresses IgE production by B cells and increases IL-10, promoting regulatory B cells. IL-10 increases IgG4, which is linked to positive outcomes after allergen desensitization immunotherapy [47]. The precursor 25(OH)D has similar effects to the active form on purified B cells, but at higher concentrations [47,48].

**Figure 4:**
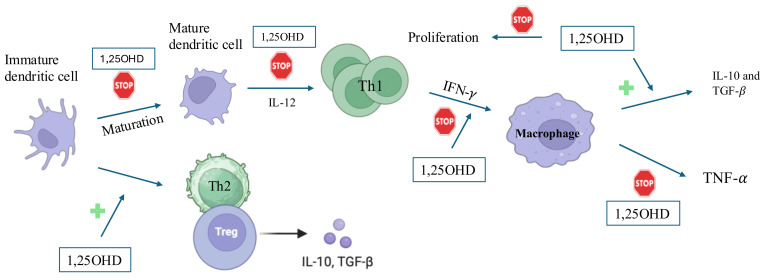
Mechanism of action of vitamin D on dendritic cells and lymphocytes. In particular, calcitriol promotes the expression of the anti-inflammatory cytokine IL-10, and it suppresses IL-12 production, which determines the differentiation of T helper cells into Th1 cells. Conversely, the anti-inflammatory cytokine IL-10 promotes the differentiation of regulatory T cells. Moreover, calcitriol promotes Th2-type differentiation and the secretion of IL-4, IL-5, and IL-10, anti-inflammatory cytokines. 1,25(OH)2D: 1-25-Dihydroxyvitamin D; IL-2: interLeukin-12; IL-10: interLeukin-10; Th1: T Helper 1 lymphocytes; Th2: T Helper 2 lymphocytes; T reg: T regulatory cells; TGF-β: transforming growth factor beta; TNF-α: Tumor Necrosis Factor alpha.

Naïve T cells express low levels of VDR, which increase upon activation. Calcitriol promotes anti-inflammatory cytokine secretion and suppresses pro-inflammatory cytokines, reducing Th1-type differentiation. Vitamin D suppresses the production of pro-inflammatory cytokines (IFNγ and TNF-α), specifically by inhibiting the p38 mitogen-activated protein (MAP) kinase pathway in monocytes [24,26,49,50,51]. Calcitriol inhibits Th17 secretion of IL-17, IFNγ, IL-21, IL-22, and granzyme B and downregulates Th17-type differentiation by suppressing IL-23 production and by acting on RAR-related orphan receptor C and aryl hydrocarbon receptor (Figure 4). Vitamin D probably acts by reducing the expression of RORγt/IL-17 by inhibiting the translocation of the p65 transcription factor to the nucleus, decreasing IL-17 mRNA levels [52,53,54,55]. Vitamin D may play a role in some inflammatory diseases, as Th17 cells are important in their pathogenesis. Conversely, calcitriol induces the FoxP3 transcription factor that promotes regulatory T cell (Treg) differentiation [26,56,57] **(**Figure 4**).** Finally, vitamin D induces the expression of CD200, an immunoglobulin-like anti-inflammatory protein, on T cells, regulating the immune response through its binding to cells expressing the CD200 receptor [3].

Regarding mast cells, vitamin D promotes anti-inflammatory IL-10 production by mast cells. It also suppresses mast cell activation, reducing histamine and TNF-α release, including IgE-dependent activation [47].

Vitamin D and its active metabolites play a significant role in endothelial function, influencing both genomic and non-genomic pathways. Specifically, 25(OH)D and calcitriol help stabilize the vascular endothelium through a non-genomic mechanism. Calcitriol interacts with the VDR, activating intracellular pathways, including adenylyl cyclase/cyclic adenosine monophosphate (AC/cAMP) and inositol trisphosphate/diacylglycerol (IP3/DAG), which leads to the upregulation of endothelial nitric oxide synthase (eNOS), which boosts the production of nitric oxide (NO) in the endothelium and enhances calcium signaling within endothelial cells. Additionally, VDR activation triggers eNOS activation via the PI3K/Akt pathway, facilitating the phosphorylation of eNOS at serine-1779 [58]. In animal models, calcitriol has been shown to stimulate endothelial cadherin-based cellular junctions, reduce the formation of stress fibers, and limit the organization of intracellular endothelial gaps, helping to prevent endothelial damage [59]. Furthermore, vitamin D metabolites play a crucial role in protecting against vascular dysfunction and systemic inflammation, both of which are key contributors to tissue injury. Vitamin D also regulates several genes involved in immunity, which are critical for understanding immune-related diseases and diagnosing vitamin D deficiency. For instance, CD93 glycoprotein plays a role in innate immunity, affecting cell adhesion, phagocytosis, and inflammation in monocytes, endothelial cells, granulocytes, platelets, and stem cells. CD93 functions as a receptor for TLR9, playing a key role in inflammatory responses through Lipopolysaccharide (LPS)/TLR4 signaling [60]. The Ninjurin1 protein, which is expressed in myeloid and endothelial cells, contributes to the immune pathogenesis of diseases such as multiple sclerosis, promoting cell adhesion and leukocyte migration, particularly during endothelial inflammation [61]. The CEBPB gene, a vitamin D target in myeloid leukemia cells, is involved in inflammation via the Th17 pathway, particularly in models of multiple sclerosis. Additionally, ACVRL1, a protein within the transforming growth factor-beta superfamily, is involved in monocyte differentiation to macrophages. Finally, SRGN, expressed by monocytes, macrophages, lymphocytes, mast cells, and endothelial cells, is involved in the secretion of inflammatory mediators following LPS exposure [62], and it is crucial in the inflammatory response and immune system modulation [63].

## 4. Allergic Diseases

Allergic diseases represent a pervasive public health concern. It has been estimated that one in four people in Western countries will become allergic [64].

An allergy is defined as an excessive immune response, categorized as a type I hypersensitivity reaction, directed towards various natural proteins, including pollen, house dust mites, food allergens, insect venom, or drugs. The allergic response is characterized by a sensitization phase, during which allergens are presented by Antigen-Presenting Cells (APCs) to naïve T cells through class II Major Histocompatibility Complex (MHC) molecules. This results in the differentiation of T cells into Th2 cells, which in turn produce IL-4 and IL-13, stimulating the production of IgE by B cells. Subsequently, in the second effector phase, following a new exposure to the allergen, IgE binds to the high-affinity IgE receptor (FcεRI) expressed by mast cells and basophils, resulting in degranulation, defined as a substantial discharge of inflammatory mediators stored in cytoplasmic secretory granules. These include, but are not limited to, histamine, leukotrienes, and heparin, which are responsible for allergic clinical manifestations. In addition, the release of IL-5 and IL-13 by T cells causes eosinophil activation and tissue infiltration, which is responsible for chronic inflammation [64,65].

Some studies have demonstrated a correlation between vitamin D and allergic diseases, including asthma, allergic rhinoconjunctivitis, atopic dermatitis, chronic urticaria, allergic contact dermatitis, and food allergies [66]. In the following paragraphs, we will analyze the possible therapeutic role of administering vitamin D in some allergic diseases, as recent clinical trials show.

### 4.1. Vitamin D, Asthma, and Rhinosinusitis

Asthma, the most common chronic respiratory disease in children, is characterized by chronic airway inflammation, remodeling, hyperresponsiveness, and airflow limitation, leading to symptoms such as wheezing, dyspnea, chest tightness, and cough, with its pathogenesis involving genetic, immunological, and environmental factors, including vitamin D status [67]. At the same time, rhinosinusitis, an inflammatory condition affecting the nasal mucosa and paranasal sinuses, presents with nasal obstruction, itching, rhinorrhea, sneezing, and ocular symptoms, affecting 10–20% of the global population and frequently associating with asthma [68]. Multiple studies demonstrate an inverse relationship between maternal and infant vitamin D levels and asthma prevalence and severity, with deficiency (<25 nmol/L) increasing exacerbation risk by 25% [69]. While definitions vary, most studies classify sufficient levels as ≥30 ng/mL (≥75 nmol/L), insufficient as 20–29.9 ng/mL (50–74.9 nmol/L), and deficient as <20 ng/mL (<50 nmol/L) [70,71,72]. Higher maternal vitamin D intake during pregnancy, particularly with levels > 30 ng/mL, reduces offspring asthma risk [73,74,75,76]. Genetic analyses suggest maternal 17q21 genotype influences this protection [77], with meta-analyses confirming lower vitamin D in asthmatic children [78] and a U-shaped dose-response relationship showing optimal protection at ~70 nmol/L but potential risk beyond ~130 nmol/L [79]. In contrast, asthmatics with low vitamin D levels exhibit reduced FEV1 and FEV1% [80]. In rhinosinusitis, deficiency (<10 ng/mL) correlates with severe allergic rhinitis and elevated IgE [81], and insufficiency (<30 ng/mL/<20 ng/mL) is a risk factor for chronic rhinosinusitis with nasal polyps [68,82,83], with in vitro studies showing vitamin D3 analogs reduce fibroblast proliferation via Regulated on Activation Normal T Expressed and Secreted (RANTES) and eotaxin suppression while enhancing cAMP expression [83]. Children with vitamin D > 75 nmol/L have lower aeroallergen sensitization. However, maternal intake or infant supplementation does not significantly alter allergic rhinitis risk, with observational data suggesting lower prevalence in those > 75 nmol/L versus < 50 nmol/L, particularly in males, though RCTs are needed [84]. Vitamin D modulates immunity by reducing Th17-driven inflammation [67,85] and urban particulate matter-induced Th17 responses [52], while supplementation may reduce rhinovirus replication, shift Th1/Th2 balance toward Th2 dominance (reducing IFN-γ, IL-5, IL-6, IL-8, and IgE while increasing TGF-β, IL-10, and IL-4) [77,79,86,87], enhance glucocorticoid responsiveness, in particular with a dose of 50,000 IU, via glucocorticoid receptor GRα upregulation [88], and inhibit remodeling by downregulating MMPs, fibronectin, and IL-6 [38,39]. Clinical trials show mixed results, with some reporting reduced exacerbations [89] but others such as the vitamin D Add On Therapy Enhances Corticosteroid Responsiveness in Asthma (VIDA) trial finding no significant benefit [90], potentially due to genetic variability, as VDR single-nucleotide polymorphisms (SNPs) such as ApaI (rs7975232), BsmI (rs1544410), FokI (rs2228570), TaqI (rs731236) and rs3782905a influence asthma susceptibility [91,92,93], while in allergic rhinitis, vitamin D with intranasal steroids improves symptom scores [like Total Nasal Symptom Score (TNSS) and Rhinitis Control Assessment Test (RACT)] and modulates CD4+/CD8+ ratios and cytokines (TNF-α↓, IFN-γ↑, IL-10↑) [94,95], and Sublingual ImmunoTherapy (SLIT) with vitamin D reduces symptoms in children, though larger trials are needed [96,97]. All these findings suggest that vitamin D supplementation may be a cost-effective, low-risk, and promising method for asthma control and symptom improvement in rhinosinusitis, both when added to intranasal steroid spray or antihistamine medication and when combined with SLIT, which in this context represents a new, effective, and well-tolerated immunotherapy modality. Further studies are needed to define this new therapeutic option.

### 4.2. Vitamin D and Atopic Dermatitis

Atopic dermatitis (AD), the most common chronic skin disease affecting 10–20% of children, is characterized by chronic pruritus and results from genetic predisposition, immune dysregulation, and impaired skin barrier function, increasing infection susceptibility [98,99,100,101]. The immune dysfunction in AD involves reduced innate immune cell activity at TLR sites, decreased IL-17 production, and impaired Treg cell function, all of which contribute to disease pathogenesis [100,101,102]. Vitamin D plays a crucial role in AD pathophysiology by enhancing cAMP expression in keratinocytes through Act1 and MEK/ERK activation, potentially explaining the therapeutic benefits of UVB therapy through vitamin D-mediated AMP production [36]. Importantly, vitamin D upregulates epidermal barrier proteins, including filaggrin, involucrin, loricrin, and transglutaminase [99], while its metabolism in skin and immune cells is regulated by Cyp27b1 and Cyp24a1 enzymes. Vitamin D also modulates immune responses by increasing Treg cell numbers and function through (DC)-mediated mechanisms involving PD-L1 expression and direct effects on CD4+ T cells to promote IL-10-secreting Tregs [102]. Clinical observations demonstrate that AD patients, particularly children, frequently exhibit vitamin D deficiency/insufficiency, with lower levels correlating with greater disease severity as measured by the Scoring of Atopic Dermatitis (SCORAD) index and elevated IL-4, IL-17A, and IL-22 levels [98,101]. Intervention studies report that daily supplementation with 1000–2000 IU vitamin D for 1–2 months significantly improves eczema scores [7,46,103,104,105,106,107], though these findings require cautious interpretation due to limitations including variable supplementation protocols, unmeasured confounders (sun exposure, diet, topical treatments), and lack of data in infants and pregnant women [108]. Neonatal vitamin D deficiency appears to increase later AD risk, while supplementation in children reduces symptoms and Th17 cell activity [101]. Genetic studies reveal associations between AD severity and polymorphisms in vitamin D metabolism genes, particularly elevated Cyp24a1 in severe cases and specific Cyp27b1 loss-of-function variants in adult subtypes [99]. While UVB therapy’s anti-inflammatory effects suggest potential for vitamin D as an oral or topical treatment [36], current evidence remains insufficient to establish standardized supplementation protocols or identify optimal candidate populations, necessitating further interdisciplinary research to translate biochemical improvements into clinically meaningful outcomes [98,107].

## 5. Vitamin D and Autoimmune Diseases

Several clinical studies have shown that vitamin D deficiency and receptor disorder/polymorphism may be associated with the development and progression of rheumatoid arthritis (RA), psoriatic arthritis (PA), SLE, multiple sclerosis, and autoimmune endocrine diseases such as Hashimoto’s thyroiditis (HT) and Graves’ disease (GD), type 1 diabetes, and Addison’s disease [2,5]. However, the role of vitamin D supplementation in treating these diseases is still unclear, as some studies have shown that vitamin D supplementation is beneficial, while others have shown no effect on key parameters of these diseases. These results may be due to differences in the type and dose of supplementation, as well as individual characteristics of the patients who participated in the studies. Therefore, new multicenter clinical trials are needed to elucidate potential underlying mechanisms [2].

### 5.1. Vitamin D and Rheumatoid Arthritis

Rheumatoid arthritis (RA) represents a complex autoimmune disorder characterized by chronic inflammation of synovial joints, leading to progressive cartilage and bone destruction. The pathophysiology of RA involves a multifaceted interplay between immune cells, inflammatory mediators, and genetic factors, with emerging evidence highlighting vitamin D’s significant immunomodulatory role.

At the molecular level, elevated IL-8 production by macrophages, neutrophils, and osteoclasts contributes substantially to the inflammatory milieu and bone resorption observed in RA [109]. This chemokine not only promotes leukocyte recruitment but also facilitates osteoclast activation, thereby accelerating joint destruction.

The immunopathogenesis of RA involves dysregulated interactions between DCs and T cells, influenced by metabolic factors such as glycolysis and reactive oxygen species (ROS), as well as vitamin D status [110].

Th17 cells in patients with early RA induce an inflammatory feedback loop for synovial fibroblast-mediated RA response interactions, including autocrine interleukin IL-17A production. The combination of 1,25(OH)2D and 1,25(OH)2D was found to regulate Th17 activity and synovial inflammation in patients with RA and potentially in patients with other Th17-mediated inflammatory diseases, suggesting that activation of VDR signaling may be more valuable than current TNF-neutralization strategies [104].

Clinically, vitamin D deficiency is prevalent in RA patients and correlates with increased disease activity (higher DAS28 scores), greater pain sensitivity by Visual Analog Scale (VAS), and reduced muscle strength [111,112,113,114].

Seasonal variations further support this relationship, as disease flares often coincide with lower vitamin D levels in winter and spring [115].

While high-dose 1,25(OH)2D therapy is limited by hypercalcemia, the non-calcemic analog 20S(OH)D3 has demonstrated efficacy in suppressing arthritis in preclinical models by activating a naturally suppressive receptor on leukemic cells called the Leukemic Immunoglobulin-1 Receptor (LAIR-1), highlighting its potential as a novel treatment. Vitamin D suppressed arthritis in LAIR-1-sufficient DR1 mice, while it was ineffective in LAIR-1^-/-^ deficient mice [116].

In patients receiving abatacept, a frequently proposed alternative to rituximab that blocks T-cell co-stimulation, 1,25(OH)2D enhanced inhibition of T-cell activators and strongly suppressed T-cell activation induced by cross-linked anti-CD3, inhibiting T-cell activation, supporting its role as an adjunct therapy [117]. Considering the results of the studies presented in Table 1, oral alfacalcidiol (1α-hydroxyvitamin D3) at a dose of 2 micrograms/day has a positive effect on disease activity in the proportion of cases showing complete remission, symptom reduction, or no new symptoms. In addition, oral alfacalcidiol improved pain control, muscle function, and bone metabolism, though longer-term data are needed to assess its impact on fracture risk [118,119]. Interesting results from four meta-analyses show that TaqI and FokI VDR polymorphisms also have a significantly significant involvement in the development of RA. In particular VDR polymorphisms rs7975232, rs1544410, rs2228570, and rs731236 were significantly associated with RA susceptibility in some populations, particularly in Asians. The contribution of the VDR was modest, confirming that RA is a complex disease and therefore multiple genes may be involved, each of which makes a minor contribution to the risk of arthritis. Further characterization of VDR, in addition to traditional and associated risk factors, may contribute to the early identification of patients at high risk of developing RA and the development of new prevention and treatment strategies [111,112,113,120].

Furthermore, alfacalcidol has shown sustained benefits in preventing glucocorticoid-induced osteoporosis, and combination therapies with hormone replacement therapy (HRT) suggest additional immunomodulatory effects by reducing serum Ig levels and B- and T-cell-independent responses to recall antigens, though further research is needed to clarify these interactions [121,122].

The evolving understanding of vitamin D’s role in RA reflects the broader shift toward personalized medicine in rheumatology. Although it is not a panacea, the rational use of vitamin D supplementation is a safe and cost-effective adjunct to conventional therapies that has the potential to improve outcomes for many patients.

The challenge for the future is to better define which patients will benefit most and under which therapeutic conditions.

Current evidence supports routine vitamin D status assessment in RA patients, with supplementation recommended for those with deficiency. However, several questions remain regarding optimal dosing strategies, long-term effects on disease progression, and the potential benefits of vitamin D analogs with improved safety profiles. Future research should focus on personalized approaches that consider genetic factors, disease stage, and concurrent medications to maximize therapeutic outcomes while minimizing adverse effects.

### 5.2. Vitamin D and Psoriasis

Psoriasis is an autoimmune skin disease that causes plaques to appear on the skin, affecting about 2–3% of the world’s population. The most common type is psoriasis vulgaris, accounting for around 80–90% of cases, while other types include pustular, gutta-percha, inversion, and erythrodermic psoriasis, affecting children and adults, respectively. Psoriatic lesions appear on the scalp, skin folds, hands, feet, nails, and genitalia [123].

Psoriasis is a multifactorial skin disease. Although the inheritance pattern is unclear, a genetic predisposition plays a pivotal role in its susceptibility. The immunopathogenesis of psoriasis involves a complex inflammatory cascade. It is initially triggered by innate immune cells (keratinocytes, DCs, natural killer (NK) cells, macrophages, fibroblasts, and γδ T cells) activated by external or internal factors. Genetically predisposed individuals are susceptible to the disease. The cytokines produced by innate cells activate myeloid DCs. They increase the production of other cytokines. These are involved in the differentiation of lymphocytes into the primary adaptive immune cells Th1, Th22, and Th17. These cells play a central role in disease pathogenesis. The cytokines produced by these cells, including TNFα, IL-22, and IL-17A/F, lead to keratinocyte proliferation. They also lead to neoangiogenesis, chemokine production, neutrophil and CD8+ cell migration into the epidermis, and a chronic inflammatory process [124]. The activity of these cells is modulated by Treg, which can inhibit the immune response and prevent autoimmune responses against self-antigens [125]. Vitamin D [126] represents a potential therapeutic factor and protective agent in psoriasis.

The effects of vitamin D on the immune system in psoriasis are complex. Vitamin D inhibits IL-17-induced expression of IL-1Ra, IL-36α, IL-36β, and IL-36γ and TNF-α-induced expression of IL-1Ra, IL-36Ra, IL-36α, IL-36γ and HBD2 in human keratinocytes. In addition, vitamin D affects T-cell homing in the skin: the expression of the chemokine receptor CXCR10 on T cells is increased by vitamin D treatment, enabling these cells to respond to the skin-specific chemokine CCL27 secreted by keratinocytes. In contrast, vitamin D reduces the expression of cutaneous lymphocyte-associated antigen (CLA) on T cells. Epidermal keratinocytes synthesize 1,25(OH)2D and are targeted by this hormone, which exerts intracrine, autocrine, and paracrine effects on keratinocytes and neighboring cells. It regulates growth, differentiation, apoptosis, and other biological processes via genomic and non-genomic effects. The non-genomic effects of 1,25(OH)2D and analogs are partly related to effects on intracellular calcium [127], whereas the genomic effects of 1,25(OH)2D are mediated by binding to the VDR. This predominant nuclear receptor protein is present in target tissues and binds 1,25(OH)2D with high affinity and low capacity [128]. As vitamin D can promote terminal differentiation and inhibit proliferation of keratinocytes [129], impairment of VDR expression in epidermal skin cells may be involved in the pathogenesis of psoriasis, and not only changes in VDR expression but also VDR polymorphism are likely to be associated with psoriasis [126].

Clinically, psoriasis patients exhibit significantly lower serum 25(OH)D levels compared to healthy controls, with the most severe cases showing the greatest deficiency [118,119,126,130,131,132,133,134,135], though oral supplementation demonstrates limited efficacy in improving Psoriasis Area and Severity Index (PASI) scores, likely due to variable dosing and bioavailability [136].

Emerging evidence indicates that moderate to severe psoriasis is inversely correlated with serum vitamin D levels. However, these levels show no significant association with arthritis, family history, age at diagnosis, or disease duration [137]. This suggests that vitamin D supplementation may help mitigate systemic inflammation and oxidative stress, potentially delaying the development of psoriasis-associated comorbidities [138]. Vitamin D analogs, including maxacalcitol, tacalcitol, and calcipotriol, have been used as adjuvants in psoriasis treatment due to their ability to regulate epidermal cell differentiation, inhibit proliferation and angiogenesis, and modulate immune responses, all with a low risk of hypercalcemia [139,140,141,142]. In murine models, topical maxacalcitol significantly reduced inflammation and MHC class II APC infiltration in imiquimod (IMQ)-induced psoriasiform skin, though this was demonstrated in a prevention rather than treatment model [53]. Calcipotriol, introduced in the late 1980s, has shown consistent efficacy and safety, with studies reporting a 50–60% reduction in PASI scores within four weeks and no significant alterations in calcium metabolism [143,144]. Combined formulations, such as calcipotriene (0.005%) with betamethasone dipropionate (0.064%), have further improved treatment outcomes. PAD^TM^ technology cream containing this combination has demonstrated high efficacy and safety in two multicenter, randomized phase 3 trials [145,146], though limitations include missing data on scalp psoriasis, adolescent populations, and long-term intermittent use. Additional studies confirmed that calcipotriol/betamethasone foam, betamethasone foam, and clobetasol propionate ointment reduce epidermal thickness and improve tPASI scores. Notably, only the CAL/BDP foam combination significantly reduced CD8⁺ IFNγ⁺ T cells and neutrophils in lesional skin, though over a short treatment duration [147]. Long-term use of tacalcitol has also proven effective. A multicenter trial using tacalcitol (4 μg/g) daily for 18 months showed sustained efficacy and safety without affecting serum calcitriol, calcium, or PTH levels [148]. A separate study using a higher dose (20 μg/g) demonstrated PASI score reduction and minor local adverse effects, with stable serum calcium homeostasis despite reduced PTH and 1,25(OH)_2_D levels [149]. A recent meta-analysis found that topical corticosteroids and vitamin D analogs offered comparable outcomes in PASI, Investigator’s Global Assessment (IGA), and Patient Global Assessment (PGA) scores, although evidence certainty was low. Common limitations included a lack of scalp psoriasis data and withdrawal due to local irritation from vitamin D analogs [150]. Importantly, a systematic review found no benefit from oral vitamin D supplementation in patients with normal baseline levels and no clear superiority among different vitamin D formulations [151]. Taken together, current evidence supports the role of topical vitamin D analogs as an effective and safe component of psoriasis management, particularly when used in combination with other therapies.

### 5.3. Vitamin D and Systemic Lupus Erythematosus

Systemic lupus erythematosus (SLE) is a chronic autoimmune disease marked by widespread immune dysregulation, including T-cell imbalance, B-cell hyperactivation, autoantibody production, and immune complex deposition, contributing to inflammation, vasculitis, and organ damage. While the precise role of vitamin D in SLE remains uncertain, whether causal or consequential, its immunomodulatory potential has drawn increasing interest. Vitamin D deficiency is prevalent in SLE patients, likely due to sun avoidance and sunscreen use, along with factors such as age, obesity, skin pigmentation, geographic location, and pharmacological treatments. Hydroxychloroquine impairs 1-α hydroxylation of 25(OH)D, while corticosteroids accelerate vitamin D catabolism, especially at doses >10–25 mg prednisone equivalent/day, reducing 1,25(OH)_2_D levels [111,152,153]. Vitamin D’s active form, 1,25(OH)_2_D, acts through the VDR, modulating immune responses by suppressing pro-inflammatory cytokines such as TNF-α, IL-17, Monocyte Chemoattractant Protein-1 (MCP-1), and supporting NK cell function [48,154,155]. Although vitamin D deficiency alone does not cause autoimmunity, it may contribute to the presence of genetic and environmental factors [115]. Some studies report lower IL-23 and higher pro-inflammatory cytokines in deficient patients, though findings vary. In addition, BsmI, FokI, and bb VDR polymorphisms have been associated with increased SLE risk, particularly in Asian populations [111,156,157]. Although anti-vitamin D antibodies have been detected in some patients, they do not consistently correlate with disease markers, except for anti-dsDNA [152,158]. In animal models (MRL/lpr and NZB/NZW), 1,25(OH)_2_D treatment reduced anti-dsDNA and anti-ssDNA antibodies, inflammatory cytokines, skin lesions, and renal pathology while improving survival effects comparable to high-dose corticosteroids [152,159]. Human studies support an association between low 25(OH)D levels and increased disease activity. The Nurses’ Health Study I and other case-control and cross-sectional studies showed lower vitamin D levels in SLE patients, particularly those with renal disease, photosensitivity, and reduced physical function [23,160,161,162]. While some inconsistencies exist, vitamin D deficiency correlates with fatigue, musculoskeletal symptoms, and lower quality of life. vitamin D supplementation has been shown to reduce disease activity measured by systemic lupus erythematosus disease activity index (SLEDAI), systemic lupus erythematosus disease activity index 2000 (SLEDAI-2K), European Consensus Lupus Activity Measurement Index (ECLAM), fatigue by fatigue severity scale (K-FSS), inflammatory markers (e.g., IL-6), flare frequency, and steroid/immunosuppressant use, although the results are not uniform [111,163,164,165,166,167,168]. Supplementation may also improve bone health and cognitive function, as well as reduce the risk of infection [152,169,170,171]. Novel strategies include combining vitamin D with tetanus toxoid to enhance immunogenicity [172] and using nonhypercalcemic analogues to avoid hypercalcemia while maintaining immunomodulatory effects [48]. Given the prevalence of hypovitaminosis D and its association with increased disease activity and comorbidities, routine monitoring and supplementation are recommended in SLE management. Optimizing vitamin D levels may help reduce disease burden, improve outcomes, and minimize long-term complications such as osteoporosis and cardiovascular disease [152,168,173].

### 5.4. Vitamin D and Autoimmune Thyroid Diseases

Hashimoto’s thyroiditis (HT) is a T cell-mediated autoimmune disorder where proinflammatory cytokines such as IFN-γ induce MHC class II HLA-DR antigen expression on thyrocytes, triggering immune-mediated thyroid damage. The active form of vitamin D, 1,25(OH)_2_D, exerts immunomodulatory effects by promoting B cell apoptosis, reducing thyroid autoantigens and antibodies, and modulating T cell and DC function, thereby protecting against thyroid autoimmunity.

A recent meta-analysis identified a significant association between the FokI VDR polymorphism and HT in Asians, but not Caucasians, while TaqI, ApaI, and BsmI showed no significant associations [174].

Several clinical studies report low vitamin D levels in HT patients. In an Indian study, weekly supplementation of 60,000 IU of vitamin D over eight weeks in deficient patients improved TSH levels and reduced antibody titers compared to placebo. However, patients with normal vitamin D levels were excluded, limiting generalizability [175].

A randomized trial also found that cholecalciferol supplementation reduced TPO-Ab and Tg-Ab levels, although the study’s small sample size, short duration, and limited demographic diversity constrained its conclusions [176]. These results are supported by three meta-analyses indicating that vitamin D3 supplementation, especially for periods >12 weeks, reduces thyroid autoantibodies and improves FT3 and FT4 levels in HT patients [116,177,178]. Despite promising outcomes, optimal dosing and treatment duration remain undefined, necessitating larger multicenter trials [175].

In addition to vitamin D, the vitamin D analog elocalcitol (BXL-628) has shown promise in modulating immune responses in thyroid cells. Compared to methimazole (MMI), elocalcitol reduced CXCL10 secretion and Th1-type cytokine activity while promoting a Th2 shift and lowering TRAb levels in Graves’ disease models. These findings highlight its potential as an immunomodulatory agent in autoimmune thyroid disorders and support exploring combination therapies to enhance efficacy and reduce side effects [179].

### 5.5. Vitamin D and Type 1 and Type 2 Diabetes Mellitus

Type 1 Diabetes Mellitus (T1DM) is a chronic autoimmune disorder characterized by immune-mediated destruction of pancreatic beta-cells (β-cells), leading to impaired insulin production or function [180]. Current knowledge suggests that an important predisposing role to the onset of the disease could be played by vitamin D: impairment of its signaling, especially in the first years of life, which increases the risk of autoimmunity [181,182,183]. Given the role that vitamin D plays in the immune system, it is believed that it may have a protective role in the development of T1DM [184].

In the pancreas of affected patients, there is an inflammatory infiltrate composed of T-cells, B-cells, and macrophages; 1,25(OH)2D promotes macrophage differentiation, which is essential in animal models of T1DM for the activation of anti-inflammatory phenotype (M1→M2) via the VDR-PPARgamma signaling pathway; it also reduces effector T-cell numbers and promotes the induction of T-reg cells [185,186,187].

The Diabetes Autoimmunity Study in the Young (DAISY) reported that increased maternal intake of vitamin D in food reduced the risk of autoimmunity against pancreatic cells in their offspring, but there is no effect of 1,25(OH)2D on residual β-cell function and insulin requirements in adults [188].

Systematic reviews and meta-analyses indicate that maintaining adequate vitamin D status in the first years of life reduces the risk of diabetes [189,190,191,192]. Hypovitaminosis D is more common in people with T1DM, and the TEDDY study reported that a higher infant concentration of 25(OH)D is associated with lower islet autoimmunity [193]. Similarly, a Finnish birth cohort study suggested that sufficient vitamin D supplementation could mitigate T1DM risk [194].

A substudy of EURODIAB demonstrated that lower serum vitamin D levels were linked to an increased risk of developing T1DM before age 15 (OR 0.63) [195]. A substudy of EURODIAB demonstrated that lower serum vitamin D levels were linked to an increased risk of developing T1DM before age 15 (OR 0.63) [196]. Human studies have also identified associations between VDR polymorphisms and T1DM risk, as well as β-cell function. Although 25(OH)D is the primary circulating form, pancreatic β-cells can convert it to 1,25(OH)2D [135]. Elevated 25(OH)D levels may serve as a substrate for local 1,25(OH)2D synthesis in β-cells, while circulating 1,25(OH)2D could exert autocrine and paracrine effects. However, vitamin D is unlikely to be beneficial after clinical diagnosis due to established β-cell damage [197]. A meta-analysis revealed that vitamin D supplementation in T1DM patients reduced daily insulin requirements and improved fasting C-peptide (FCP), stimulated C-peptide (SCP), and HbA1c levels [198]. While some studies disagree [199], numerous observational studies support a strong association between vitamin D deficiency and T1DM [14,200].

Type 2 Diabetes Mellitus (T2DM) is a chronic metabolic disorder characterized by inadequate insulin production and, consequently, hyperglycemia.

Vitamin D deficiency or insufficiency is implicated in both macrovascular and microvascular complications of T2DM through multiple mechanisms. Macrovascular complications, such as endothelial dysfunction, arterial stiffness [201,202], peripheral arterial disease, and carotid plaque formation [203], are exacerbated by hypovitaminosis D. Microvascular complications, including diabetic retinopathy, neuropathy, and nephropathy, are also influenced by vitamin D status. For instance, vitamin D deficiency may worsen retinopathy severity by affecting angiogenesis and immune cell activity [204] and impair nociceptor function in neuropathy [205]. In addition, VDR is also expressed in highly insulin-sensitive tissues (pancreas, adipose tissue, and muscle) [206], and vitamin D works like an epigenetic factor mediating the transcription level and enhancing insulin sensitivity [180].

In line with recent studies, vitamin D supplementation is implicated in plasma HbA1c reduction, suggesting that vitamin D can contribute to reducing the development of diabetic complications [196]. Additionally, supplementation has been shown to improve β-cell function [207] and insulin sensitivity [206,208,209,210], particularly in high-risk individuals.

In pancreatic tissue, vitamin D protects β-cell function by the activation of the VDR expressed in β-cells, reducing local inflammation and increasing insulin secretion [211,212,213]. Vitamin D may play a role in the transcriptional activation of a gene contained in the human insulin receptor and in regulating the opening and closure of calcium channels, which regulates insulin secretion, mediating the calcium flux in β-cells, and interacting with receptors (VDR and 1,25D3-MARRS). Therefore, vitamin D deficiency, causing an alteration in calcium flux, could interfere with normal insulin secretion [214,215]. Increasing circulating vitamin D concentration could affect tissue energy and metabolism, improving systemic insulin sensitivity. The skeletal muscle is crucial in insulin sensitivity, involving 70–90% of total glucose disposal during the postprandial period [216,217,218]. Thus, vitamin D supplementation might improve skeletal muscle glucose handling and, as a consequence, insulin sensitivity [219]. Vitamin D also regulates the adipose tissue, and hypovitaminosis may play a role in obesity and fat mass due to the restoration of vitamin D, a fat-soluble vitamin, in the adipose tissue [220]. Hypovitaminosis also seems to increase microvascular complications such as diabetic retinopathy, diabetic neuropathy, diabetic nephropathy, and diabetic foot ulcers [221], and a meta-analysis demonstrated that increased circulating vitamin D levels protect the kidney from injury and ameliorate proteinuria in T2DM patients [197].

## 6. Vitamin D and Infectious Diseases

The immune system’s defense against pathogens involves a coordinated response between innate and adaptive immunity [198]. The innate immune system is activated to neutralize the pathogen when it encounters an infectious agent. Subsequently, the adaptive immune response is initiated, typically taking 5–7 days to eliminate remaining pathogens. If the adaptive response fails, the innate system intensifies its activity, releasing pro-inflammatory cytokines [199,200]. Elderly and chronically ill individuals often exhibit an enhanced innate response, potentially due to immuno-inflammatory dysregulation associated with cellular aging, increasing their susceptibility to cytokine storms [201]. The NLRP3 inflammasome, a critical component of the innate immune system, promotes the production of IL-1β and IL-18, which stimulate downstream cytokines such as IL-6 [137,202]. Its activation is implicated in COVID-19 pathogenesis and cytokine storms, with associated markers such as CRP and LDH indicating inflammation [203]. Oxidative stress, characterized by excessive reactive oxygen species (ROS) and impaired detoxification, is a known trigger for NLRP3 activation, resulting in decreased immune recognition and ineffective pathogen clearance [204]. Vitamin D modulates inflammasome activation. While it inhibits NLRP3, it promotes NLRC4 activation, which is involved in bacterial intracellular immunity [205]. Studies have shown a link between adequate vitamin D levels and reduced inflammatory markers such as IL-6 and CRP [222,223,224]. Moreover, calcitriol has demonstrated protective effects against upper respiratory infections, including influenza [225,226]. Vitamin D also interacts with the Renin-Angiotensin-Aldosterone System (RAAS), which regulates immune responses, blood pressure, and metabolism. Angiotensin II (Ang II), a primary effector, can promote inflammation and oxidative stress via the AT1 receptor pathway. Excessive RAAS activation reduces ACE2 activity, contributing to acute lung injury and ARDS [227,228,229,230,231,232]. SARS-CoV-2 binds ACE2, impeding Ang II conversion to its anti-inflammatory counterpart Ang (1–7), exacerbating inflammatory damage [233,234,235]. Vitamin D, through its active form 1,25(OH)_2_D, downregulates RAAS by suppressing renin and enhancing ACE2 expression, mitigating Ang II-induced damage [236,237,238]. This action reduces oxidative stress and inflammation, potentially alleviating lung injury, as shown in LPS-induced models [237,239]. Chronic deficiency, however, may dysregulate RAAS, promoting lung fibrosis [240]. Adequate vitamin D increases ACE2 and Ang (1–7) levels, supporting vascular and immune stability [241,242,243]. By tightening epithelial junctions and reducing pro-inflammatory cytokines, vitamin D deficiency is a key risk factor for severe COVID-19 outcomes [244,245,246,247]. Vitamin D also supports antimicrobial defenses through the regulation of autophagy and T-cell differentiation. It stabilizes VDR proteins, enhancing immune signaling and reducing proteasomal degradation [248,249]. Deficiency correlates with increased susceptibility to respiratory pathogens and elevated inflammatory cytokines such as IL-6 and IL-8, with NF-κB inhibition observed upon supplementation [250,251,252]. While evidence suggests vitamin D reduces sepsis-related inflammation, clinical findings are mixed. Some trials indicate a reduction in RTI incidence and antibiotic use, especially with lower daily doses (≤1000 IU), though effects on asthma or COPD exacerbations remain inconclusive [253,254,255,256,257]. Concerning COVID-19, meta-analyses of randomized controlled trials (RCTs) do not show consistent results. While vitamin D supplementation did not significantly reduce mortality, it was associated with reduced ICU admissions and ventilator use. Observational studies link low 25(OH)D levels to increased COVID-19 risk, severity, and mortality [258,259]. In VAP patients, supplementation improved serum vitamin D and reduced procalcitonin, although no significant effect on SOFA scores was observed—likely due to limited sample size and duration [260]. Another RCT in healthcare workers found that high-dose vitamin D, an oral bolus of 100,000 IU with 10,000 IU/week of vitamin D, rapidly raised serum levels and may confer immunomodulatory benefits, although the trial ended prematurely [261].

### Vitamin D and Acquired Immunodeficiency Syndrome (AIDS)

Recent studies have shown the role of vitamin D in regulating host-bacteria interactions, and its supplementation may reduce inflammation and HIV complications [41]. Vitamin D supplementation stimulating the expression of cAMP improves antibacterial immunity in monocyte cultures and HIV plasma [44] and is associated with reduced bacterial DNA translocation in HIV/HCV-coinfected patients. Low vitamin D levels are linked to high plasma HIV viral load, decreased CD4+ T-cells [262,263], faster-progressing AIDS, and lower survival in HIV patients [264,265]. High concentrations of vitamin D can boost autophagy activity, suppressing HIV replication in white cells [266].

Vitamin D deficiency may play a role in the development of HIV and could contribute to the progression of the infection by weakening the immune system. Low vitamin D levels can increase the risk of inflammation, immune system activation, and non-AIDS-related complications in people with HIV.

A potential benefit of vitamin D supplementation may be particularly relevant for children and adolescents with HIV. Low levels of vitamin D reduce the immune system’s ability to fight off infections such as Mycobacterium tuberculosis, the main cause of AIDS deaths in many regions worldwide. Some authors defined vitamin D supplementation in HIV-infected children and adolescents as safe and effective. They found that a dose of 100,000 IU every 2 months, together with 1 g/day of calcium, increased serum vitamin D concentrations significantly [267].

Vitamin D is linked to a higher risk of HIV-related health problems and death, so taking extra vitamin D might stop HIV from replicating, improve tuberculosis (TB) inflammation, and reduce the progression of HIV-TB co-infection. Some authors investigated the role of vitamin D in their meta-analysis, showing that HIV-TB patients had a higher prevalence of vitamin D deficiency than HIV patients, but supplementation had no effect on CD4 count or viral load [268]. Bone health is also compromised in people with HIV infection, and vitamin D supplementation may have beneficial effects on bone health. A recent meta-analysis found that vitamin D supplementation can improve serum 25(OH)D in HIV-infected patients; BMD and PTH need further investigation on a larger scale [269]. The current evidence highlights the relationship between vitamin D status and disease progression, demonstrating both potential promise and significant unanswered questions. While observational data consistently associate deficiency with worse outcomes, intervention studies reveal more nuanced results. These limitations suggest that while vitamin D assessment and repletion may be reasonable in HIV care, its role as a disease-modifying intervention requires substantially more evidence before widespread clinical implementation can be recommended.

## 7. Vitamin D and Multiple Sclerosis

Multiple sclerosis (MS) is a chronic autoimmune disorder affecting over 2 million people, predominantly young adults. It involves inflammation and demyelination in the central nervous system, impacting myelin, oligodendrocytes, axons, and neurons [270]. Clinically, MS manifests with paresthesias, ataxia, cognitive deficits, and impairments in vision and mobility [271]. While its etiology remains unclear, MS is associated with over 200 genetic loci [272] and environmental risk factors, notably Epstein-Barr virus infection, vitamin D insufficiency, and smoking [273]. Although the optimal serum 25(OH)D level is debated [269], levels above 40 ng/mL are considered protective in MS [274]. Sun exposure during childhood and its interaction with VDR gene variants may influence MS risk [275,276]. Polymorphisms in the VDR gene have been linked to MS, although findings remain inconsistent [277]. The FokI variant, which alters VDR protein structure, is associated with higher transcriptional activity [278,279], while other polymorphisms may affect RNA stability and VDR binding, with potential implications for immune regulation [280,281,282]. These genetic variations may increase MS susceptibility, particularly under conditions of vitamin D deficiency [283,284]. Geographical latitude and vitamin D levels also appear to correlate with MS prevalence [273]. Low serum 25(OH)D is associated with increased MS risk, disease activity, and progression [285,286,287]. Patients with MS tend to have lower vitamin D levels compared to the general population [288], and some studies link higher 25(OH)D levels with better disability outcomes [289]. Evidence on the efficacy of vitamin D supplementation in MS is mixed. A meta-analysis of 12 studies (950 patients) examined outcomes such as annualized relapse rate (ARR), Expanded Disability Status Scale (EDSS), and MRI lesion activity. Results showed a modest increase in ARR with high-dose vitamin D (mean difference 0.15; 95% CI 0.01–0.30) and a non-significant trend toward EDSS improvement (mean difference −0.12; 95% CI −0.78 to 0.01) [290]. However, high-dose supplementation did not outperform low-dose, and findings did not consistently support clinical benefit, highlighting the need for further placebo-controlled trials. Another meta-analysis of 12 RCTs (933 participants) found no significant effects of vitamin D (mostly D3) on ARR, EDSS, or gadolinium-enhancing lesions over 52 weeks [287]. Outcomes related to relapse timing, hospitalization, cognition, and psychological symptoms were not reported, limiting conclusions. An additional review of 10 RCTs on vitamin D (10 to 98,000 IU/day) found mixed results. While most trials showed no clinical benefit, some reported reduced IL-17 and increased IL-10 and TGF-β cytokines, along with improvement in active lesions and EDSS in patients with low baseline vitamin D [291,292]. Importantly, no adverse effects were reported. A study on pregnant women with MS and low 25(OH)D levels (<20 ng/mL) found that weekly supplementation with 50,000 IU of vitamin D3 improved serum levels (mean 33.7 ng/mL vs. 14.6 ng/mL, *p* < 0.05) and was associated with fewer relapses during pregnancy and up to six months postpartum. EDSS remained stable in the intervention group but worsened in the control group (*p* < 0.07) [293]. No significant adverse events were noted. Lastly, the D-Lay MS trial [294], a parallel, double-blind, randomized, placebo-controlled clinical trial including 316 individuals aged 18 to 55 years, provides compelling evidence for vitamin D’s therapeutic potential in early MS. High-dose cholecalciferol (100,000 IU every 2 weeks) significantly reduced disease activity in patients with clinically isolated syndrome (CIS), as measured by relapse rates and MRI lesions. These findings align with vitamin D’s known immunoregulatory properties, supporting its role as a potential adjunct therapy in neuroinflammatory disorders, significantly reducing disease activity in CIS and early relapsing-remitting MS.

In conclusion, while vitamin D is implicated in MS risk and may have immunomodulatory effects, current evidence from clinical trials does not conclusively support its use to improve clinical outcomes. Further large-scale, well-controlled studies are warranted to clarify its role in MS management.

## 8. Conclusions

The growing body of evidence highlights the multifaceted role of vitamin D in modulating immune responses and its potential impact on immune-mediated diseases. In Table 1, we provide a detailed summary of the findings investigating the effectiveness of vitamin D supplementation, including specific dosage recommendations for the diverse conditions addressed in this review. While preclinical and observational studies consistently demonstrate immunomodulatory properties of vitamin D, such as promoting regulatory T cell development, inhibiting pro-inflammatory cytokines, and enhancing innate immunity, translating these findings into clinical practice remains complex. Critically, the association between vitamin D deficiency and increased susceptibility to autoimmune and infectious diseases, including MS, SLE, T1DM, HIV, and COVID-19, is compelling but not conclusive. Much of the current evidence is correlative, and interventional studies show heterogeneous results. This suggests that while vitamin D supplementation may offer therapeutic or preventive benefits in deficient individuals, it is unlikely to serve as a universal remedy. Furthermore, variability in study design, dosing regimens, baseline vitamin D status, genetic polymorphisms affecting vitamin D metabolism, and disease heterogeneity complicate the interpretation of results. A one-size-fits-all approach is, therefore, inappropriate. Future research should prioritize large-scale randomized controlled trials with standardized protocols and stratified analyses to clarify the contexts in which vitamin D supplementation is most effective.

In conclusion, although maintaining adequate vitamin D status appears prudent, given its pleiotropic biological roles, current evidence does not support widespread therapeutic use in immune-mediated diseases. Further studies are necessary to provide more sophisticated, personalized approaches that go beyond blanket supplementation strategies to target specific pathological mechanisms in defined patient subgroups. Vitamin D remains an intriguing immunomodulator, but its clinical utilization requires much more rigorous investigation.

**Table 1:** Synthesis of different clinical studies on the effectiveness of various dosages of vitamin D administration in pathologic conditions analyzed.

Author	Design	Duration	Participants (CA/CO)	Dose of Vitamin D	Results
**VITAMIN D AND ASTHMA**					
Majak et al. [295]	RCT	6 months	48 children (aged 5 to 18 years) with new diagnoses of asthma (24/24)	Participants were randomly assigned to: - Budesonide 800 mg/day administered as a dry powder and vitamin D placebo or - Budesonide 800 mg/day administered as a dry powder and 500 IU vitamin D cholecalciferol	The vitamin D group showed a significantly lower number of children who experienced asthma exacerbation and a lower number of children with a decrease of 25(OH)D than the steroid group (*p* = 0.029), so vitamin D supplementation prevented the reduction of serum concentrations of 25(OH)D and reduced the risk of asthma exacerbation triggered by acute RTIs.
Nanzer et al. [296]	RCT	4 weeks	24 patients (aged 18 to 75 years) with glucocorticoid-resistant asthma (13/11)	In the screening period, patients received a 2-week course of oral prednisolone; after a 4-week washout period, patients were randomly divided into two groups that received calcitriol 0.25 μg soft capsules or placebo twice daily for 4 weeks with a second course of oral prednisolone identical to the first repeated during the final 2 weeks.	The calcitriol group showed a modest but significant improvement in absolute and predicted FEV1 compared to the placebo group (*p* = 0.03), so 1,25(OH)2D supplementation improved the clinical response to oral steroids.
Castro et al. [90]	RCT	28 weeks	408 adults (aged 18 years or older) with asthma and a serum 25(OH)D level of less than 30 ng/mL (201/207)	Participants were randomly assigned to either a placebo or a high-dose of vitamin D (100,000 IU once), followed by 4000 IU/day for 28 weeks added to inhaled ciclesonide (320 µg/d; 2 puffs twice/day) and levalbuterol; subsequently, inhaled corticosteroids were tapered by 50% if the participant’s asthma symptoms were controlled.	Treatment with vitamin D did not alter the rate of first treatment failure or exacerbation compared with the placebo group; however, among secondary outcomes, the only statistically significant one was a small difference in the overall dose of ciclesonide required to maintain asthma control in the vitamin D group compared to the placebo group (111.3 µg/d [95% CI, 102.2–120.4 µg/d] in the vitamin D group vs. 126.2 µg/d [95% CI, 117.2–135.3 µg/d] in the placebo group; difference of 14.9 µg/d [95% CI, 2.1–27.7 µg/d]).
Mahboub et al. [88]	RCT	8 weeks	54 adults (aged 18 to 65 years) with moderate-to-severe asthma and 25(OH)D levels less than 20 ng/mL (34/20)	Participants were randomly assigned to: - The experimental group received 50,000 IU of vitamin D orally weekly - The placebo group received the placebo	There was a significant increase in GR-α expression in the experimental group compared to the placebo control (*p* < 0.05), while no change in GR-β expression was observed; consequently, the GR-α/GR-β ratio increased significantly (*p* < 0.05).
Wang Q. et al. [78]	MT (12 RCTs)	From 6 weeks to 6 months	1871 children with asthma (898/973)	Participants were randomly assigned to: - The experimental group received vitamin D supplements (from 200 IU/day to 60,000 IU/month) - The placebo group received the placebo	The experimental group had a significantly lower recurrence rate than the placebo group (18.4% versus 35.9%, RR = 0.35, 95% CI = 0.35–0.79, *p* = 0.002).
Wang M. et al. [297]	MT (14 RCTs)	From 3 to 12 months	1421 patients with asthma (711/710)	Four studies compared vitamin D (from 500 IU/day to 50,000 IU/week) to placebo as a treatment individually, while other studies received vitamin D as an adjunct treatment (corticosteroids/SABA/LABA/Montelukast/conventional therapy not described).	Vitamin D supplementation was associated with: - a significant improvement of FEV1% in patients with vitamin D insufficiency and airflow limitation (baseline FEV1% < 80%) (MD: 8.30, 95% Cl (5.95, 10.64) - a reduction in the rate of exacerbation compared with placebo (RR: 0.7395% Cl (0.58,0.92)).
Wang Y. et al. [77]	MT (19 RCTs)	From 6 weeks to 48 weeks	2063 patients with asthma (1039/1024)	Participants were randomly assigned to: - The experimental group received vitamin D supplements (from 100 IU/day to 60,000 IU/week) - The placebo group received placebo	In the vitamin D supplementation group: -Number of exacerbations was less, while there was no statistical difference (OR = 0.73, *p* = 0.06, I^2^ = 59%) - The FEV1/FVC was significantly improved (OR = 4.33, *p* = 0.02, I^2^ = 99%) - Length of hospital stay and mortality were not changed - Levels of IL-5 and IgE were significantly decreased (OR = −9.18, *p* = 0.0004, I^2^ = 99%; OR = −100.85, *p* < 0.00001, I^2^ = 0%) - Levels of IL-6 and IL-10 and eosinophil counts were not significantly different, while in subgroup analysis based on serum vitamin D, the IL-10 level of the vitamin D deficiency group was significantly increased (OR = 2.51, *p* < 0.00001, I^2^ = 32%).
Fedora et al. [86]	MT (10 RCTs)	From 6 weeks to 12 months	1243 asthmatic patients from 0 to 18 years old (631/612)	Participants were randomly assigned to: - The experimental group received vitamin D supplements (from 150 IU/day up to 4000 IU/day) - The placebo group received placebo	In the vitamin D supplementation group there was: - A significant reduction of asthma exacerbations (RR 0.62; 95% CI: 0.44, 0.87; *p* = 0.006) (primary outcome) - A significant improvement in predicted percentage of FEV1 levels
Pojsupap et al. [298]	MT (5 RCTs)	From 4 to 52 weeks	625 asthmatic patients (from 5 to 18 years old)	Participants were randomly assigned to: - The experimental group received vitamin D supplements (from 500 IU/day up to 2000 IU/day) - The placebo group received placebo or budesonide ICS	In vitamin D supplementation group, there was a statistically significant reduction in asthma exacerbation (RR 0.41, CI 0.27–0.63).
Jolliffe et al. [89]	MT (7 RCTs)	From 15 weeks to 1 year	955 asthmatic patients	Participants were randomly assigned to: - The experimental group received vitamin D supplements (from 500 IU/day up to 2000 IU/day) - The placebo group received placebo	Vitamin D supplementation reduced the rate of asthma exacerbation requiring treatment with systemic corticosteroids among all participants (adjusted incidence rate ratio [aIRR] 0.74, 95% CI 0.56–0.97; *p* = 0.03). There were no significant differences between vitamin D and placebo in the proportion of participants with at least one exacerbation or time to first exacerbation.
Niu et al. [299]	MT (12 RCTs)	From 9 weeks to 9 months	1295 asthmatic patients (649/646)	Participants were randomly assigned to: - The experimental group received vitamin D supplements (from 400 IU up to 100,000 IU) - The placebo group received placebo	Vitamin D supplementation significantly reduced the number of asthma exacerbations, including the rate of exacerbations requiring systemic corticosteroid therapy and the rate of acute exacerbations requiring emergency department or hospital visits or both.
Riverin et al. [300]	MT (8 RCTs)	From 1 to 12 months	573 asthmatic children aged 3 to 18 years	Participants were randomly assigned to: - The experimental group received vitamin D supplements (from 1000 IU/week up to 60,000 IU/month) - The placebo group received placebo, budesonide, fluticasone, or prednisone	In vitamin D supplementation group there were: - a reduced risk of asthma exacerbations (RR 0.41, 95% CI 0.27 to 0.63, 3 studies, n = 378). - no significant effect for asthma symptom scores and lung function.
Chen et al. [292]	MT (12 RCTs)	From 2 to 12 months	1543 asthmatic patients (769/774)	Participants were randomly assigned to: - The experimental group received vitamin D supplements (from 1000 IU/week up to 60,000 IU/week) - The placebo group received placebo	Vitamin D supplementation significantly reduced the risk of asthma exacerbation (pooled risk ratio (RR) 0.70, 95% confidence interval (CI), 0.59, 0.83; *p* < 0.05), but did not improve ACT score or lung function among patients with asthma treated with corticosteroids.
**VITAMIN D AND ALLERGIC RHINITIS**					
Bhardwaj et al. [94]	RCT	2 years and 5 months	87 patients (aged 16 to 60 years) with allergic rhinitis and serum vitamin D levels less than 20 ng/mL	Participants were randomly divided into two groups: - Group A received Fluticasone 50 mcg nasal spray, two puffs BID for 4 weeks - Group B received Fluticasone 50 mcg nasal spray, two puffs BID, and Oral vitamin D cholecalciferol 60,000 IU once a week for 4 weeks	The pre-treatment TNSS score in Group A was 12.5 ± 2.68, while the post-treatment score was 8.98 ± 1.009, with the difference in both scores of Group A as 3.52 (the t score was 117.387 and *p* < 0.000). The pre-treatment TNSS score in Group B was 11.64 ± 3.09 while the post-treatment score was 6.3 ± 1.45, with the difference in both scores of Group B as 5.34 (the t score was 131.1403 and *p* < 0.0001). The post-treatment RCAT in Group A and Group B was 19.72 ± 2.84 and 28.2 ± 1.53, respectively, with the difference between the two groups as 8.48 (t score as 135.27 and *p* < 0.05).
Guo [95]	RCT	5 years	140 patients (aged 16 to 60 years) with moderate-to-severe allergic rhinitis (70/70)	Participants were randomly divided into two groups: - The control group received 200 µg mometasone nasal spray and two puffs of BID for 4 weeks. - The experimental group received 200 µg mometasone nasal spray, two puffs BID, and two oral vitamin D capsules (400 IU vitamin D per capsule) for 4 weeks.	Total TNSS scores were significantly reduced compared with those before treatment in both groups for all four main symptoms assessed (*p* < 0.05), and the improvements with vitamin D supplementation were significantly greater than those of the control group in the symptoms assessed, except for sneezing (*p* < 0.05). RQLQ scores in both groups were significantly lower after the therapy than before the therapy in all aspects (*p* < 0.05). The improvements with vitamin D supplementation were significantly higher than those in the control group, except for eye symptoms, which were improved. Still, the difference was not statistically significant compared to the control (*p* < 0.05).
Bakhshaee et al. [96]	RCT	1 year	80 patients (aged 18 to 40 years) with allergic rhinitis and vitamin D level of 10–20 ng/mL (40/40)	Participants were randomly divided into two groups: - The control group received a placebo plus cetirizine for eight weeks - Intervention group received vitamin D (weekly 50,000 IU) plus cetirizine for eight weeks	There was no significant difference between the two groups at the onset of the study and 4 weeks later regarding the mean scores of symptom severity (*p* = 0.073), whereas a significant difference was obtained between the two groups in terms of symptom severity at baseline and 8 weeks post-treatment (*p* = 0.007).
Jerzynska et al. [97]	RCT	5 months	50 children (aged 5 to 12 years) with grass-related moderate-to-severe rhinoconjunctivitis; eight patients had concomitant asthma (25/25)	Participants were randomly divided into two groups: - The control group received SLIT with a placebo - The experimental group received SLIT with vitamin D 1000 IU daily supplementation	The experimental group shows a reduction of nasal symptoms (*p* = 0.04), asthma symptoms (*p* = 0.001), and combined symptom-medication score (*p* = 0.001); there was no significant difference between groups in medication and ocular scores.
**VITAMIN D AND ATOPIC DERMATITIS (AD)**					
Kim et al. [46]	MT (11 studies: 7 observational trials and 4 RCTs)	In observational trials: N/A; in clinical trials: from 60 days to 2 months	In observation trials, 1643 children and adults with AD or healthy subjects (986/657); in clinical trials, 194 children and adults with AD (104/90)	In observational trials: N/A; in clinical trials: from cholecalciferol or ergocalciferol 1000 IU daily to cholecalciferol 1600 IU daily vs. placebo	In observational trials, the AD group had lower serum 25(OH)D levels compared to the healthy group (statistically significant in the pediatric AD patients, not statistically significant in the adult AD group); in clinical trials, the SCORAD index or EASI score decreased significantly (mean difference: −5.85 points) in the vitamin D group.
Hattangdi-Haridas et al. [103]	MT (16 studies: 11 observational studies and five interventional studies, including RCT, not RCT, clinical intervention, and audit trials)	In observational trials: N/A; in interventional trials: from 60 days to 3 months	In observational trials: adults and children or adults only or children only with AD or healthy subjects (1221/982); in interventional trials: adults and children with AD (165/170)	In observational trials: N/A; in interventional trials: cholecalciferol 1000 IU/daily or 1600 IU/daily or 2000 IU/daily	In observational trials, the AD group had a statistically significantly lower vitamin D concentration compared to the healthy group (*p* = 0.02); in interventional trials, for the repeated measures interventions, the SCORAD index decreased significantly (mean difference: 21 points; *p* < 0.0001) in vitamin D group; for the randomized control trials, the SCORAD index reduced significantly (mean difference: 11 points; *p* < 0.0001) in the vitamin D group.
Huang et al. [105]	SR (21 articles: 4 RCTs, 5 cohort studies, 6 case-control studies, 6cCross-sectional studies; vitamin D supplementation was investigated in 6 studies: 4 RCTs and 2 cohort studies)	From 4 to 12.86 weeks	A total of 354 patients with an average age of 6.79 years; participants involved with an RCT are 227 (114/113).	Vitamin D doses ranged from 1000 to 2000 IU/daily; cholecalciferol was used in 2 articles, while 2 studies were supplemented with ergocalciferol, and the remaining 2 studies did not specify what type of vitamin D was used.	Significant improvement of AD when supplemented with vitamin D was found in 67% (4/6) of studies.
Nielsen et al. [107]	MT (12 RCTs)	From 4 to 12 weeks	686 patients with AD (347/339)	From 1000 to 5000 IU/daily and 8000 to 60,000 IU/weekly; cholecalciferol was predominantly used, except for one study that utilized ergocalciferol.	Significant improvement in AD symptoms following vitamin D supplementation was found in 27% (3/11) of studies in the intervention group compared with the control group.
**VITAMIN D AND RHEUMATOID ARTHRITIS (RA)**					
Andjelkovic et al. [301]	RCT	3 months	19 patients treated with standard DMARD therapy for acute RA	Oral alfacalcidol (2 micrograms/day)	Positive effect on disease activity in 89% of the patients: - 45% or nine patients with complete remission - 44% or eight patients with a satisfactory effect - 11% no improvement, but no new symptoms occurred
Scharla et al. [121]	RCT	4 weeks	71 inpatients with RA and osteopenia	Patients were randomly assigned to: - Group 1 treated with 1000 IU plain vitamin D + 500 mg calcium daily - Group 2 treated with 1 µg of alfacalcidol + 500 mg calcium daily	In patients with RA, alfacalcidol, but not plain vitamin D, improved calcium and bone metabolism, modulated inflammation, improved muscular function, and decreased pain symptoms.
Forsblad et al. [122]	RCT	2 years	88 women with RA aged between 45 and 65 years	All patients were treated with a daily dose of 400 IU vitamin D; - 41 women were randomized to receive HRT (oestradiol and noretisterone acetate), vitamin D and calcium - 47 to the control group receiving vitamin D and calcium alone	There are no differences between the HRT and control groups concerning the concentration of Ig in serum and to B- and T-cell-dependent recall antigen reactions. Thus, HRT did not stimulate nor suppress these immunological responses.
**VITAMIN D AND PSORIASIS**					
Pinter et al. [146]	MT (2 Phase 3 RCTs)	8 weeks	1271 patients: 783 male and 488 female subjects above the age of 18 years with mild-to-moderate psoriasis according to PGA and with a treatment area of 2–30% of the body (trunk and/or limbs), with a PASI as a baseline of at least of 2 or 3	Subjects were randomly allocated to one of three arms: - 551 for CAL/BDP PAD-cream; - 178 for cream vehicle; - 542 for active comparator [a marketed CAL/BDP gel/topical suspension. The number of subjects who completed the trial was, 526 (for CAL/BDP PAD cream), 149 (for cream vehicle), and 513 (for active comparator) respectively.	CAL/BDP PAD cream demonstrated superiority after 8 weeks compared to CAL/BDP TS for all efficacy endpoints, including PGA treatment success, mPASI, and PASI75. Superiority was also demonstrated for patient-reported DLQI and PTCS.
Heim et al. [147]	RCT	4 weeks	30 patients (18 male and 12 female subjects) with mild psoriasis defined by a PASI score below 10 and at least two symmetric lesions of 2 cm^2^ affecting the knees or the elbows.	Subjects were randomly allocated into three groups of ten. All patients applied calcipotriol/betamethasone foam to their elbows on one side of the body. Three groups of 10 patients applied either placebo foam (emollient), betamethasone foam or clobetasol propionate ointment to elbows on the opposite side of their body. The side of the treatment allocation was randomly assigned using central randomization.	All treatments, including placebo, provided a significant decrease in tPASI. The treatment with calcipotriol/betamethasone foam was the most effective in decreasing CD8+ cell infiltrate in both the dermis and epidermis after 4 weeks of treatment. They noticed that CD8+ IFNγ+ T cells decreased significantly after both betamethasone and combined calcipotriol/betamethasone treatment but also that calcipotriol/betamethasone significantly reduced the number of MPO+ neutrophils which were predominantly IL-17+
Venegas-Iribarren et al. [150]	MT (eight SRs including 26 studies overall, of which 22 were RCTs)	N/A	4238 adult patients (15 to 90 years) with plaque psoriasis in the trunk and limbs, not including the scalp and with low, moderate, or severe disease.	All trials used topical corticosteroids as intervention (fluocinonide 0.05% twice a day, betamethasone dipropionate 0.05% once or twice a day, betamethasone 17-valerate 0.1% once or twice a day, desoxymethasone 0.25% twice a day, clobetasol propionate 0.05% twice a day, and diflorasone diacetate 0.05% twice a day). As a comparison, topical treatment with calcipotriol 50 mcg/g once or twice a day, calcitriol 3 mcg/g twice a day, and tacalcitol 4 mcg/g once a day.	There might be little or no difference in PASI, IAGI, and PAGI scores between topical corticosteroids and topical vitamin D analogues. The certainty of the evidence is low. Topical corticosteroids lead to fewer local adverse events (skin irritation) and fewer withdrawals than topical vitamin D analogues. With a high certainty of the evidence. No studies were found that evaluated the impact of topical corticosteroids and topical vitamin D analogues in cutaneous atrophy.
**VITAMIN D AND SYSTEMIC LUPUS ERYTHEMATOSUS (SLE)**					
Andreoli et al. [163]	RCT	24 months	34 patients (18/16)	Participants were randomly assigned to: - Group 1: Standard Regimen (SR) = 25,000 IU/month- Group 2: Intensive Regimen (IR) = 300,000 IU bolus + 50,000 IU/month for one year and then switched to the other regimen in the second year.	After 12 months, values above 30 ng/mL were found in 75% of Intensive Regimen patients, while only 28% were in the Standard Regimen. No significant differences in disease activity and SLE serology were found between SR and IR at any time. No changes in the mineral metabolism were observed.
Lima et al. [167]	RCT	24 weeks	40 patients with juvenile-onset SLE (20/20)	Participants were randomly assigned to: - Group 1: cholecalciferol 50,000 IU/week - Group 2: placebo	SLEDAI score and ECLAM significantly improved (*p* = 0.011, *p* = 0.006) Fatigue score and K-FSS significantly improved (*p* = 0.012, *p* = 0.008) No significant improvements in cutaneous and articular manifestations and proteinuria (*p* = 0.66, *p* = 0.18, *p* = 0.32).
Karimzadeh et al. [164]	RCT	3 months	90 patients (with levels of 25(OH)D < 30 ng/mL) (45/45)	Participants were randomly assigned to: - Interventional group patients: vitamin D3 soft gel capsules (50,000 IU/weekly for 12 weeks and then 50,000 IU/monthly for 3 months) - Placebo group: placebo	The mean of vitamin D levels significantly increased in the interventional group (*p* < 0.001) Not significantly different in SLEDAI score before and after vitamin D administration (*p* = 0.39).
Singgih Wahono et al. [165]	RCT	14 months	40 patients	Participants were randomly assigned to: - Group I: 3 × cholecalciferol 400 IU and 3 × 1 tablet placebo - Group II: 3 × cholecalciferol 400 IU and curcumin *(Curcuma xanthorrhiza)* 3 × 20 mg	Supplementation of cholecalciferol 1200 IU either with placebo (*p* = 0.000) or with curcumin (*p* = 0.003) significantly increased serum 25(OH)D levels. Serum vitamin D levels differed significantly, with higher levels in group I (*p* = 0.047). The delta of vitamin D levels (the difference between vitamin D levels after supplementation and before supplementation) did not differ significantly between the two groups (*p* = 0.166). SLEDAI scores significantly decreased in both groups (Group I: *p* = 0.001); Group II: *p* = 0.003) Serum IL-6 levels significantly decreased in both groups (Group I: *p* = 0.001); Group II: *p* = 0.013).
Pakchotanon et al. [166]	RCT	24 weeks	104 patients (52/52)	Participants were randomly assigned to: - Group A: ergocalciferol 100,000 IU weekly for 4 weeks, followed by ergocalciferol 40,000 IU weekly for 20 weeks, - Group B: placebo	The mean standard deviation of serum levels of vitamin D in group A was significantly higher than in group B (*p* < 0.001). There is no difference between groups A and B concerning SLEDAI-2K, flare events, ESR, CRP, and dosage of immunosuppressive drugs.
**VITAMIN D AND AUTOIMMUNE THYROID DISEASES**					
Zhang et al. [302]	MT (8 RCTs)	From 4 to 24 weeks	652 patients with HT (332/320)	Participants were randomly assigned to: - Vitamin D (in different formulations and dosages) ± LT4 - LT4/Sunshine and diet/placebo	Vitamin D supplementation: - Significantly decrease TPO-Ab levels in the subgroups in whom the treatment duration > 3 months (*p* = 0.009) and in the subgroups treated with vitamin D3 (*p* = 0.006) - Was effective in reducing the Tg-Ab levels (*p* = 0.009) (due to limited data, no subgroup analysis and meta- regression analysis were performed).
Wang S. et al. [177]	MT (6 RCTs)	From 1 to 6 months	344 patients with AIT (178/166)	Participants were randomly assigned to: - Vitamin D (in different formulations and dosages) ± LT4/elemental calcium 500 mg/day - LT4/elemental calcium 500 mg/day/placebo/no treatment	Vitamin D supplementation significantly decrease TPO-Ab levels at six months (3 RCT, *p* < 0.01) and significantly decrease Tg-Ab levels (4 RCT, *p* = 0.033; even if a significant heterogeneity was found) No significant effect on changes in TSH, FT3, or FT4 after vitamin D supplementation.
Tang et al. [178]	MT (12 RCTs)	From 12 to 24 weeks	862 patients with HT (429/423)	Participants were randomly assigned to: - Vitamin D (in different formulations and dosages) ± LT4 - LT4/placebo/no treatment	Vitamin D supplementation: - Significantly decrease TPO-Ab levels (*p* = 0.03) - Significantly increase fT3 and fT4 levels (*p* < 0.001) at 12 weeks - Significantly decrease Tg-Ab levels (*p* < 0.001; even if a significant heterogeneity was found) -Significantly decreases TSH levels
**VITAMIN D AND TYPE 1 AND TYPE 2 DIABETES MELLITUS**					
Gregoriou et al. [303]	MT (7 RCTs)	From 4 to 52 weeks	287 patients with T1DM	Participants were randomly assigned to: - Calcitriol 0.25 μg per day or on alternate days plus insulin - Alphacalcidol 0.5 μg daily plus insulin cholecalciferol 2000 IU per day plus insulin for 18 mo - Cholecalciferol 70 IU/kg body weight/day plus insulin	Vitamin D supplementation in the form of alphacalcidol and chole-calciferol appears to be beneficial in DID, FCP, SCP, and HbA1c.
Najjar et al. [304]	MT (10 studies: 3 cohort; 5 case–control; 2 matched case–control)	N/A	39,884 patients with T1DM (16,370/23,514)	N/A	No large effect of a genetically determined reduction in 25(OH)D concentrations by selected polymorphisms on T1DM risk.
Nascimento et al. 2022 [305]	RS (10 studies)	From 6 to 52 weeks	Children and adolescents (0–19 years) with T1DM	- Cholecalciferol with dosages ranging from 1000 to 160,000 IU. - Just one study used vitamin D in the form of alfacalcidol at a dosage of 0.25 to 0.5 μg/day	This study did not provide evidence to support the effect of vitamin D supplementation on glycemic control to aid in the treatment of T1DM.
Yu et al. [306]	RS (13 studies: 9 RCTs; 2 open-label case–control, 1 open label, and 1 cohort	From 4 to 12 weeks	527 patients with T1DM	The following therapeutic regimens were used: 1.25 D 0.25 μg twice daily; 25D 2000 IUdaily; 25D to achieve serum 25 D > 125 nmol/L; alfacalcidol 0.25 μg BD 25 D; 60,000 IU monthly; Ergocalciferol (D2) 2 m of 50,000 IU/w; 25D 2000 IU/d; 25D 3000 IU/d; Calciferol 2000 IU/d + etanercept + GAD-alum	The maintenance of optimal circulating 25 D levels may reduce the risk of T1DM, and that may have potential for benefits in delaying the development of absolute or near-absolute C-peptide deficiency.
Hu et al. [196]	MT (19 RCTs)	From 4 to 24 weeks	1374 patients with T2DM (747/627)	- Up to 50,000 UI/weekly vitamin D3 - 300,000 UI single injection of vitamin D3	Significant reduction in HbA1c, IR (marked by a decrease in HOMA-IR) and insulin levels in the short-term vitamin D supplementation group.
Krul-Poel et al. [219]	MT (23 RCTs)	From 4 to 52 weeks	1797 patients with T2DM: for the effect on HbA1c, 1475 patients (755/720); for the effect on FBG 1180 patients (608/572)	- From 1000 IU/day vitamin D3 to 45,000 IU/week - Vitamina D3 or 1200 IU/day - Vitamin D3 for 2 weeks followed by 5600 IU/day for 10 weeks or from 100,000 to 300,000 IU - Vitamin D3 single dose	Significant effect on FBG in a subgroup of studies (n = 4); no significant effect on change in HbA1c.
**VITAMIN D AND INFECTIOUS DISEASES**					
Bergman et al. [254]	RCT	12 months	140 patients with antibody deficiency disorder and increased susceptibility to RTIs (70/70)	Participants were randomly assigned to: - Group 1: cholecalciferol 4000 UI daily for 12 months - Group 2: placebo	The overall infectious score was significantly reduced for patients allocated to the vitamin D group compared with the placebo group.
Lehouck et al. [256]	RCT	12 months	182 patients with moderate to very severe COPD and a history of recent exacerbations (91/91)	Participants were randomly assigned to: - Group 1: 100,000 IU of vitamin D supplementation every 4 weeks for 1 year - Group 2: placebo	High-dose vitamin D supplementation in a sample of patients with COPD did not reduce the incidence of exacerbations. In participants with severe vitamin D deficiency at baseline, supplementation may reduce exacerbations. (rate ratio, 0.57 [CI, 0.33 to 0.98]; *p* = 0.042).
Slow et al. [255]	RCT	3 years	135 patients were admitted to the hospital with CAP (mean baseline plasma 25(OH)D concentration was 48.7 ± 21.6 (SD) nmol/L) (68/67)	Participants were randomly assigned to: - Group 1: An oral single dose of vitamin D 20,000 UI - Group 2: placebo	Vitamin D supplementation had a significantly greater effect on those with low baseline vitamin D (<50 nmol/L) in improving the radiological resolution of pneumonia.
Miroliaee et al. [260]	RCT	10 months	51 patients with VAP who were suffering from vitamin D deficiency (26/25)	Participants were randomly assigned to: - Group 1: received 300,000 units of intramuscular vitamin D. - Group 2: placebo	The levels of PCT were significantly decreased (*p* = 0.001) in the treatment group (–0.02 ± 0.59 ng/mL) compared to that of the placebo group (0.68 ± 1.03 ng/mL), no significant changes in SOFA and CPIS scores (*p* = 0.37 and *p* = 0.46, respectively).
Arpadi et al. [267]	RCT	12 months	56 children with HIV and vitamin D levels insufficiency (29/27)	Participants were randomly assigned to: - Group 1: HIV-infected children and adolescents who were aged 6 to 16 years receive vitamin D (100,000 IU bimonthly) and calcium - Group 2: double placebo	No group differences were seen in the change in CD4 count or CD4% or viral load. The overall mean monthly serum 25(OH)D concentrations were higher in the group that received vitamin D.
Hosseini et al. [261]	RCT	16 weeks	34 patients (healthcare workers as a primary prevention of SARS-CoV-2 infection) (19/15)	Participants were randomly assigned to: - Group 1: 100,000 IU vitamin D oral bolus at randomization followed by a weekly dose of 10,000 IU of vitamin D3 - Group 2: placebo	The groups which received vitamin D supplementation (95% CI) were in favor of supplementation; 77.8% of intervention, and 13.3% of control, patients were vitamin Dsufficient (OR:6.11, 95% CI:1.6, 22.9).
Anitua et al. [257]	MT (65 RCTs)	From 5 days to 3 years	50,554 participants	All trials used vitamin D as intervention as dose ranging from 400 to 1000 IU/day	The incidence of RTIs in terms of count data (OR: 0.87; 95%CI [0.80–0.95]; *p* = 0.0028; I^2^ = 43%) and event rate (IRR: 0.81; 95%CI [0.70–0.95]; *p* = 0.010; I^2^ = 79%) was significantly reduced in the intervention group. However, no effect of vitamin D on duration or upper RTIs severity was observed.
Petrelli et al. [259]	MT (43 observational trials)	N/A	612,601 patients	All trials used an intervention group with vitamin D supplementation (from 1000 UI daily to 80,000/100,000 IU every 2–3 months or 80,000 IU within a few hours of the diagnosis of COVID-19).	Risk of COVID-19 infection was higher compared to those with replete values (OR = 1.26; 95% CI, 1.19–1.34; *p* < 0.01). Vitamin D deficiency was also associated with worse severity and higher mortality than in non-deficient patients (OR = 2.6; 95% CI, 1.84–3.67; *p* < 0.01 and OR = 1.22; 95% CI, 1.04–1.43; *p* < 0.01, respectively).
Meng et al. [258]	MT (25 RCTs)	From 7 days to 1 year	8128 participants	Preventing effect group - Group 1: 1949 participants received vitamin D supplementation, with daily dosages ranging from 800 IU to 5000 IU daily or weekly. - Group 2: 3703 participants received a placebo. Regarding the therapeutic effects - Group 1: 1270 participants received vitamin D supplementation - Group 2: 1206 participants did not receive vitamin D supplementation or received low dosage of vitamin D	The studies included in our analysis mainly administered a single-dose, high-dosage vitamin D supplementation (from 800 IU to 5000 IU) therapy for severe SARS-CoV-2 infection patients. Single-dose supplementation was found to reduce the rate of mechanical ventilation, while the multiple-dose therapy was found to reduce the rate of ICU admission and mortality.
**VITAMIN D AND MULTIPLE SCLEROSIS (MS)**					
McLaughlin et al. [290]	MT (12 interventional studies)	6 to 22 months	950 patients	Studies were divided into four groups because of heterogeneity in study design. Three studies only included patients with serum 25(OH)D levels below 50, 75, or 100 nmol/L The effective daily dosage of cholecalciferol (or equivalent ergocalciferol) used ranged from 2857–10,400 IU in the active or high-dose arms. The low-dose arms ranged from 800 to 1000 IU.	Non-significant trends in favor of vitamin D for all outcome measures: high-dose vitamin D has worse outomes in ARR (mean difference 0.15 [95% CI 0.01–0.30]) and non-significant trends of increased EDSS (mean difference −0.12 EDSS points [95% CI −0.78) to 0.01] and gadolinium-enhancing lesions for the higher-dose arms.
Jagannath et al. [287]	MT (12 RCTs)	From six months (24/26 weeks) to 12 months (48/52 weeks)	933 participants	Participants were enrolled in two groups: 464 were randomized to the vitamin D group, and 469 to the comparator group.	Vitamin D has no effect on ARR: the mean in the intervention group was 0.05 lower (−0.17 lower to 0.07 higher); worsening of EDSS: the mean score in the intervention group was 0.25 lower (0.61 lower to 0.10 higher); or new MRI gadolinium-enhancing T1 lesions: in the intervention group was 0.02 higher (0.45 lower to 0.48 higher).
Berezowska et al. [291]	MT (10 RCTs)	From 2012 to 2018	627 participants	All studies evaluated the use of vitamin D supplementation (ranging from 10 to 98,000 IU), comparing it to a placebo or low-dose vitamin D. The duration of the intervention ranged from 12 to 96 weeks.	One trial demonstrated that vitamin D supplementation resulted in fewer new T2 lesions (a mean of 0.5 compared to a mean of 1.1 in the placebo group). Four of ten studies showed no significant differences between the vitamin D and control groups. One trial found that the EDSS score increased significantly (*p* < 0.01) in a placebo group from a mean of 1.7 to 1.94, with no significant difference in scores at the end of the trial between intervention and control groups (*p* > 0.05).
Etemadifar et al. [293]	RCT	12 to 16 weeks of gestation till delivery	15 participants	Participants were randomly allocated into two groups: - Group 1: 50,000 IU/week vitamin D - Group 2: routine care from 12 to 16 weeks of gestation till delivery	Average serum 25(OH)D level at the end of the trial in vitamin D supplemented group was higher than routine care group (33.7 ng/mL vs. 14.6 ng/mL, *p* < 0.050). In the vitamin D group, the mean EDSS did not change 6 months after delivery (*p* > 0.050), whereas in the routine care group, the mean EDSS increased from 1.3 (0.4) to 1.7 (0.6) (*p* < 0.070).
Thouvenot et al. [294]	RCT	From July 2013 to December 2020 (final follow-up on January 2023)	316 participants	Patients were randomized 1:1 to receive oral cholecalciferol 100,000 IU every 2 weeks for 24 months: - Group 1: 163 participants - Group 2 placebo: 153 participants	In group 1 vs. the placebo group: MRI activity (89 patients [57.1%] vs. 96 patients [65.3%]; HR, 0.71 [95% CI, 0.53–0.95]; *p* = 0.02), new lesions (72 patients [46.2%] vs. 87 patients [59.2%]; HR, 0.61 [95% CI, 0.44–0.84]; *p* = 0.003), and contrast-enhancing lesions (29 patients [18.6%] vs. 50 patients [34.0%]; HR, 0.47 [95% CI, 0.30–0.75]; *p* = 0.001). The vitamin D oral supplementation significantly reduces the disease activity in CIS and relapsing-remitting MS.

CA/CO: Case/Control; RCT: randomized controlled trial; IU: International Unit; *p*: *p*-value; RTIs: Respiratory Tract Infections; GR: glucocorticoid receptor; AD: atopic dermatitis; MT: meta-analysis; N/A: Not Applicable; FEV1: Forced Expiratory Volume in the first second; SABA: Short Acting Beta-adrenoceptor Agonists; LABA: Long Acting Beta-adrenoceptor Agonists; ICS: Inhaled CorticoSteroids; ACT: Asthma Control Test; BID: Bis In Die; TNSS: Total Nasal Symptom Score; RACT: Rhinitis Control Assessment Test; RQLQ: Rhinoconjunctivitis Quality of Life Questionnaire; SLIT: Sublingual Immunotherapy; HRT: hormone replacement therapy; mPASI: modified (excluding the head) Psoriasis Area and Severity Index; PGA: Physician’s global assessment; PASI75: at least 75% reduction in mPASI from Baseline; PTCS: Psoriasis Treatment Convenience Scale; SCORAD: Scoring of Atopic Dermatitis; EASI: Eczema Area and Severity; SR: Systematic Review; RA: rheumatoid arthritis; HRT: Hormon Replacement Therapy; CAL/BDP PAD: calcipotriol and betamethasone dipropionate cream based on PAD™ Technology; CAL/BDP TS: calcipotriol and betamethasone dipropionate topical suspension/gel; DLQI: Dermatology Life Quality Index; IAGI: Investigators Assessment of overall Global Improvement, PAGI: Patient Assessment of Global Improvement; SLE: systemic lupus erythematosus; SR: Standard Regimen; IR: Intensive Regimen SLEDAI-2K: SLE Disease Activity Index 2000; SLEDAI: SLE Disease Activity Index; ECLAM: European Consensus Lupus Activity Measurement; K-FSS: Krupp Fatigue Severity Scale; ESR: Erythrocyte Sedimentation Rate; CRP: C-Reactive Protein; HT: Hashimoto Thyroiditis; anti-TPO: anti-Thyroid PerOxidase; LT4: levothyroxine; AIT: autoImmune thyroiditis; DID: Daily Insulin Dose; FCP: Fasting C- Peptide; SCP: Stimulated C-Peptide; HbA1c: emoglobin A1c; T1DM: Type 1 Diabetes Mellitus; T2DM: Type 2 Diabetes Mellitus; IR: insulin resistance; HOMA-IR: Homeostatic Model Assessment of Insulin Resistance; FBG: Fasting Blood Glucose; RTIs: Respiratory Tract Infections; COPD: Chronic Obstructive Pulmonary Disease; CAP: Community-Acquired Pneumonia; VAP: Ventilator-associated Pneumonia; PCT: procalcitonin; SOFA: Sequential Organ Failure Assessment; CIPS: Critical Illness Polineuropathy; HIV: Human Immunodeficiency Virus; ICU: Intensive Care Unit; multiple sclerosis (MS); ARR: annualized relapse rate; EDSS: Expanded Disability Status Scale; MRI: Magnetic Resonance Imaging; CIS: clinically isolated syndrome.
